# Molecular machineries and physiological relevance of ER-mediated membrane contacts

**DOI:** 10.7150/thno.51871

**Published:** 2021-01-01

**Authors:** Shiyin Lin, Tian Meng, Haofeng Huang, Haixia Zhuang, Zhengjie He, Huan Yang, Du Feng

**Affiliations:** 1 Affiliated Cancer Hospital and Institute of Guangzhou Medical University, Guangzhou Municipal and Guangdong Provincial Key Laboratory of Protein Modification and Degradation, School of Basic Medical Sciences, Guangzhou Medical University, 511436, Guangzhou, China.; 2 State Key Laboratory of Respiratory Disease, Guangzhou Medical University, 511436, Guangzhou, China.; 3 Department of Pulmonary and Critical Care Medicine, Hunan Provincial People's Hospital, The First Affiliated Hospital of Hunan Normal University, Changsha 410021, China.

**Keywords:** MAM, MCS, Mitochondria, ER, Autophagy, Lipid droplet, Golgi, MVB, Endosome

## Abstract

Membrane contact sites (MCSs) are defined as regions where two organelles are closely apposed, and most MCSs associated with each other via protein-protein or protein-lipid interactions. A number of key molecular machinery systems participate in mediating substance exchange and signal transduction, both of which are essential processes in terms of cellular physiology and pathophysiology. The endoplasmic reticulum (ER) is the largest reticulum network within the cell and has extensive communication with other cellular organelles, including the plasma membrane (PM), mitochondria, Golgi, endosomes and lipid droplets (LDs). The contacts and reactions between them are largely mediated by various protein tethers and lipids. Ions, lipids and even proteins can be transported between the ER and neighboring organelles or recruited to the contact site to exert their functions. This review focuses on the key molecules involved in the formation of different contact sites as well as their biological functions.

## Introduction

The largest organelle in cells is the endoplasmic reticulum (ER), a continuous single membrane-bound network that is made up of smooth tubules and rough sheets 1, 2. In different cell types, the ER has distinct shapes, but its function is similar. The ER is the major Ca^2+^ reservoir, where the concentration of Ca^2+^ is thousands of times higher than that in the cytosol 3. Thus, it is considered the primary site for providing Ca^2+^ to other organelles to maintain their essential physiological roles with the help of calcium channels, ryanodine receptors or inositol 1,4,5-trisphosphate receptors (IP3R) 4. The ER also acts as a hub of lipid synthesis, such as triacylglycerols, sphingolipids, sphingosine and ceramide intermediates, and phosphatidylserine (PS) 5. Some lipids are transferred to other organelles to be directly utilized or to be further processed and modified. Additionally, the ER is involved in the folding and processing of new proteins to carry out initial quality control processes; these proteins are subsequently delivered to the Golgi for modification and secretion 6-8. These functions depend on the efficient and fast exchange of materials between the ER and other organelles.

**Figure 1:**
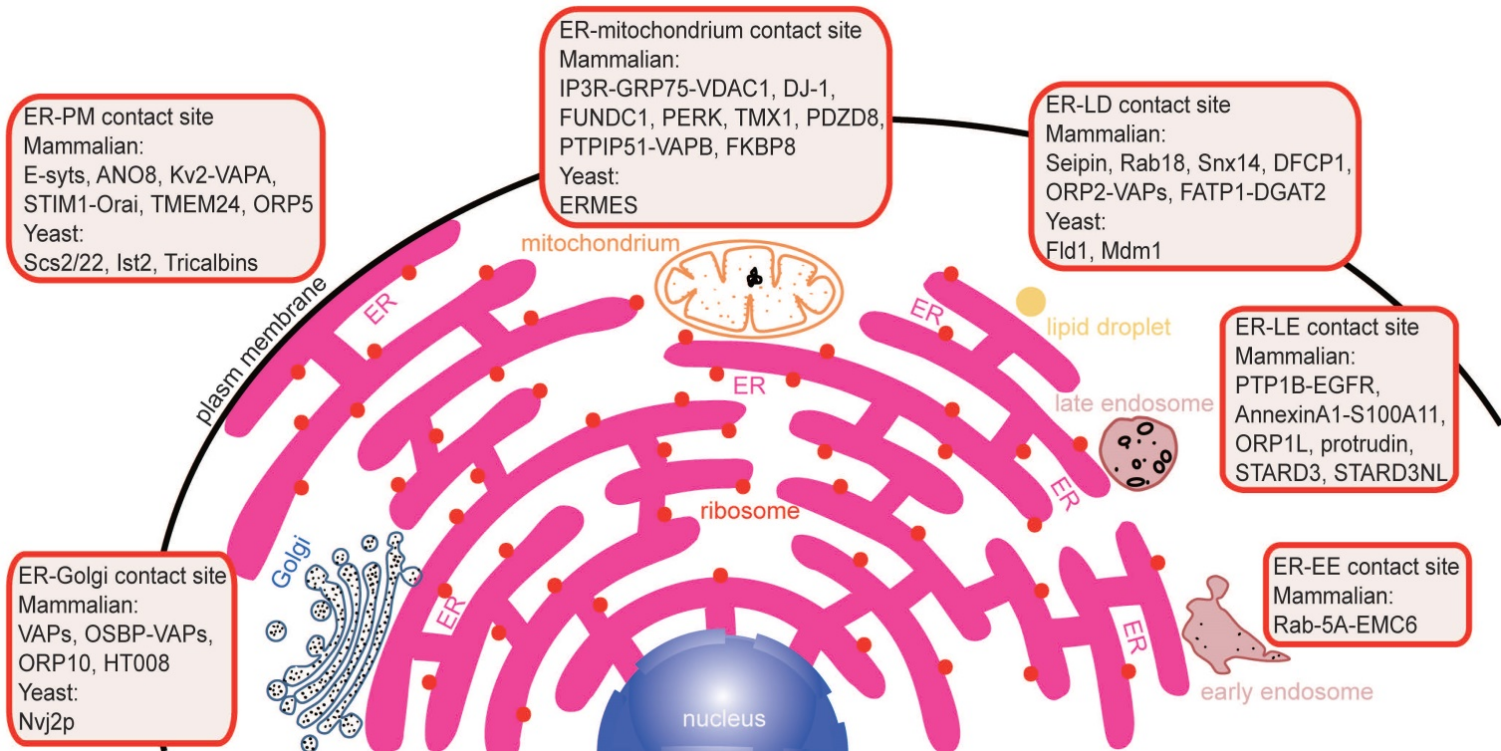
** An overview of established potential tethers at various membrane contact sites in mammals and yeasts.** A large number of proteins are involved in the formation of different membrane contact sites by protein-protein interactions or protein-lipid interactions. Overexpression or deletion of these proteins perturbs the formation of different membrane contact sites by different pathways and results in heterogeneous phenotypes.

Membrane contacts between different membrane-bound organelles can be seen clearly under an electron microscope. Membrane contact sites (MCSs) are defined as regions where two organelles are closely apposed, and their membranes are sometimes associated, often with a gap of less than 30 nm 9. At these points, substances are capable of exchanging rapidly via different mechanisms. Because the ER is distributed throughout the cell, it has extensive communication with other intracellular membrane structures, such as the plasma membrane, mitochondria, Golgi, endosomes and lipid droplets (LDs) (Figure 1). Tethers are proteins that are localized at MCSs and that support the formation of ER-mediated membrane contacts, ion transport, and organelle dynamics. Potential tether proteins for the formation of ER-mediated membrane contacts in mammals are listed in Table 1. Membrane contact is an expanding area in cell biology, and progress has been discussed in numerous excellent recent reviews 10-15. This review focuses on the major molecular machinery involved in the formation of membrane contact sites and how they coordinate to maintain the functions of these contacts (Figure 1).

## ER-PM contact sites

**Figure 2:**
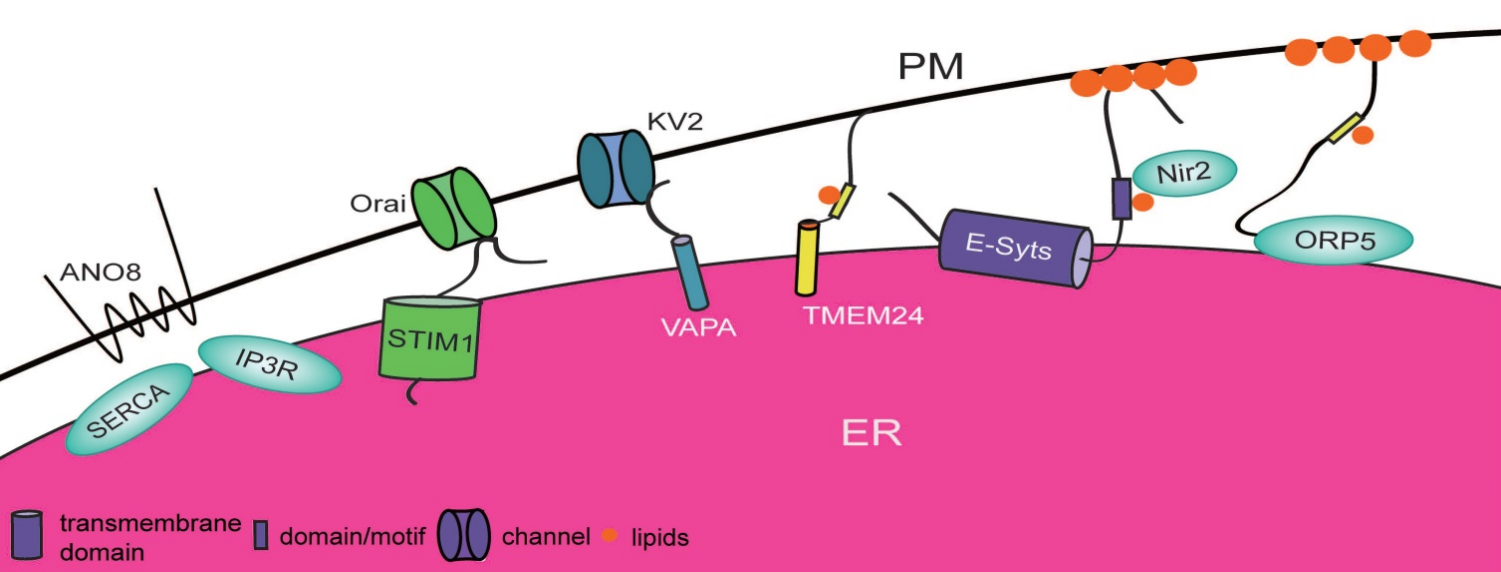
** Proteins involved in ER-PM contact sites in mammals.** Proteins that localize at either the ER or the plasma membrane shorten the distance between the ER and plasma membrane by establishing physical interactions or by their own function. Each protein plays a different role. STIM1-Orai in the ER-PM contact sites is able to mediate Ca^2+^ exchange. E-syts and ORP5 interact with lipids on the PM and anchor at the ER by their distinct domains to promote lipid transfer between these two organelles. TMEM24, ANO8, and KV2-VAPs also participate in the formation of ER-PM contact sites.

The plasma membrane is the primary protective barrier of cells. It mediates communication between the intracellular and extracellular environments via selective secretion of cytokines, absorption of nutrients, or the export of waste to maintain the growth and homeostasis of cells. There are many different ion channels located on or within the PM, including Ca^2+^-permeable ion channels and voltage-gated K^+^ channels. Distinct ion gradients are formed through ion channels; these gradients are necessary for generating appropriate membrane potential and driving action potential in excitable cells or for mediating a variety of essential cellular processes, including fertilization, hormone secretion, muscle contraction, gene transcription and cell death^16^. The phospholipid PtdIns(4,5)P_2_ (phosphatidylinositol 4,5-bisphosphate) and its precursor PtdIns4P (phosphatidylinositol 4-phosphate) are also abundant at the PM, where they assist the function of ion channels and mediate signal transduction and cell motility 17. It is worth mentioning that excitation-contraction units were first observed in muscle by means of electron microscopy in the 1950s, when the concept of ER-PM contact sites was first proposed 18. Subsurface cisterns have subsequently been described in neurons 19. ER-PM contact sites, with gaps ranging from 10 to 30 nm between the two membranes, are ubiquitous structures in many cell types with different shapes 20 (Figure 2). These contacts allow certain molecules such as proteins and lipids that are targeted to the ER or PM to establish a bridge for regulating a number of functions, such as transporting Ca^2+^ and lipids, maintaining ER morphology and enhancing neuronal excitability.

In yeast, it has been reported that Scs2, Scs22, Ist2, and tricalbins form a hierarchy in the formation of ER-PM contact sites, of which Scs2 and Scs22 are most important 21.

The VAMP (vesicle-associated membrane protein)-associated proteins Scs2 and Scs22 in yeast are localized at the ER 22. Their mammalian homologs belong to the VAP (vesicle-associated membrane protein-associated protein) family. It has been documented that an Scs2 Scs22 double mutant together with deletion of the three tricalbins has the same level of ER-PM contact as does the Scs2 Scs22 double mutant alone. However, the triple mutant Scs2 Scs22 ist2 has a significantly reduced level of ER-PM contact compared to that the Scs2 Scs22 double mutant 21. Both Scs2 and Scs22 have N-terminal MSP (major sperm protein) domains with FFAT (diphenylalanine in an acidic tract) binding capability 22, enabling them to bind to lipid-associated proteins, thereby regulating phospholipid synthesis and metabolism 23.It has been speculated that Scs2 may interact with Osh proteins, oxysterol-binding protein homologs, to promote the formation of ER-PM contact sites and thereby control PtdIns4P levels at the PM 24.

Both Ist2 (increases sodium tolerance protein 2) and three tricalbins (Tcb1p, Tcb2p and Tcp3p) are anchored at the ER 25, 26. Although mutations in Ist2 or triple tricalbins in wild-type cells do not affect ER-PM contact substantially, they aggravate ER-PM dissociation in mutant Scs2 and Scs22 cells 21, 27. Hence, Ist2 and tricalbins are considered potential candidates for connecting the ER to the PM. It has been reported that Tcbs may promote lipid exchange between the ER and the PM by forming a highly curved peak at the cortical ER. The SMP domain and C2 domain of Tcbs are necessary for this process. Moreover, lipid flows may compensate for lipid consumption at the PM to maintain the integrity of the plasma membrane upon stress 28.

In mammals, E-syts are the homologs of tricalbins. E-syt2 and E-syt3 are able to directly bind to PtdIns(4,5)P_2_ at the PM via their C2C domain under normal conditions, while E-syt1 is likely to function in the increase in cytosolic Ca^2+^ 29, 30. However, this differs in yeast; dramatic reduction in ER-PM contact sites is observed in response to triple knockdown of all E-syts, and the phenotype could be rescued by overexpression of the E-syts 29 (Figure 2).Notably, upon Ca^2+^ release, E-syt1 is able to rearrange ER structures into ring-shaped MCSs to improve the stability of ER-PM contact sites and promote the replenishment of Ca^2+^ in the ER 29, 31. Hence, E-syts contribute to the formation and stability of ER-PM contact sites in mammals to control the Ca^2+^ flux between the ER and the PM. Surprisingly, ER-PM contact site-positioned E-syts also mediate lipid transfer between the ER and the PM. The SMP domain is a lipid-binding module, and SMP-containing proteins are usually present in MCSs 32. The SMP domain of E-syts allows the nonpreferential binding of glycerophospholipids 33. E-syts also have the ability to recruit Nir2, a phosphatidylinositol transfer protein that delivers PA (phosphatidic acid) to the ER and in turn transfers PI (phosphatidylinositol) to the PM and to ER-PM contact sites upon receptor stimulation 30. It has been revealed that E-syts at ER-PM contact sites not only are involved in Ca^2+^ regulation but also control lipid transfer and promote PtdIns(4,5)P_2_ replenishment following receptor stimulation.

Recently, on the basis of sequence analysis, members of the ANO (anoctamin) family were suggested to be homologous to Ist2 in humans, but only ANO8 has the ability to tether the ER and the PM. ANO8 may control Ca^2+^ flux by assembling Ca^2+^ signaling complexes, such as STIM1 (stromal interaction molecule 1), SERCA2 (sarcoplasmic/endoplasmic reticulum calcium ATPase 2), and IP3R, at ER-PM junctions, which affects the efficiency and fidelity of cell signaling in HEK293 cells 34 (Figure 2).

The Kv2 voltage-gated K^+^ channel is a classic potassium channel 35. This protein is present in two different forms. One form localizes at the PM dispersively and acts as a voltage-dependent K^+^ channel involved in electric activity. The other form occurs at ER-PM contact sites but loses its ability to mediate electric activity. The C-terminal PRC (proximal restriction and clustering) domain of Kv2 plays a dominant role in clustering behavior rather than ion channel function. Experiments have shown that the PRC domain of Kv2 is able to interact with the FFAT-binding domain of the ER protein VAPA (vesicle-associated membrane protein-associated protein A) 36, 37. Knockdown of Kv2 or a point mutation within its PRC domain impairs the ER-PM contact sites in neurons, and depletion of VAPA also impedes the clustering behavior of Kv2 35, 36. This strongly suggests that the nonconductive form of Kv2 cooperates with VAPA to promote the organization of ER-PM contact sites, which contributes to exocytosis 38, 39 (Figure 2). Recent research has shown that Kv2.1 is able to recruit Nir2 to ER-PM contact sites and influences PI homeostasis in a VAP-dependent pathway in neurons. This suggests a novel role of the Kv2-VAP interaction in the modulation of neuronal lipid signaling and homeostasis 40.

SOCE (“Store-operated” Ca^2+^ entry) is an essential mechanism for regulating the loading of Ca^2+^ after depletion of stored Ca^2+^. It has been discovered that the STIM1-Orai complex is one of the important components of SOCE. STIM1is an important ER-resident calcium storage sensor with an ER-resident domain (including an EF-hand-binding domain and a SAM (sterile alpha-motif) domain) and a cytoplasmic portion (including an ERM (ezrin-radixin-moesin) domain, a C-terminal inhibitory domain, a serine/proline domain and a polybasic lysine-rich domain or K domain) to carry out its function. In resting cells, STIM1 is distributed uniformly at the ER by binding to Ca^2+^ in the ER lumen via its EF-hand-binding domain 41. However, once Ca^2+^ is released, STIM1 tends to cluster at the ER near the PM with the help of its SAM domain (Figure 2). At that time, the cytoplasmic domain of STIM1 starts to recruit Orai to ER-PM contact sites and induces a new interaction between the ER and the PM 42, 43. Orai is a component of the CRAC (Ca^2+^release-activated Ca^2+^) channel at the PM and can be activated by Ca^2+^ release to mediate Ca^2+^-selective flux 44. Loss of Ca^2+^ induces a conformational change in the SOAR (STIM1-Orai1 activating region) in the ERM domain of STIM1, and the exposed SOAR is able to interact with the C-terminal coiled-coil region of Orai 45. This protein-protein interaction brings the ER very close to the PM and then activates SOCE to replenish the stored Ca^2+^, maintain Ca^2+^ homeostasis, and further trigger Ca^2+^ signaling and gene transcription 46, 47.

SOCE is one of the most important processes that occurs at ER-PM contact sites, and many regulatory molecules involved in these processes have been revealed. For example, STMATE (STIM-activating enhancer), encoded by TMEM110, is a multitransmembrane protein at the ER. As its name suggests, STMATE plays a positive role in the redistribution of STIM1. The depletion of STMATE causes STIM1 to lose its ability of clustering at the ER, which further reduces the formation of ER-PM junctions in stimulated cells and suppresses the activation of SOCE and Ca^2+^ signaling. This means that STMATE has the ability to regulate the stability of ER-PM contact sites and Ca^2+^ signal transduction 48, 49. Septin is also a positive regulator in HeLa cells and promotes both the interaction of STIM1 with Orai and the recruitment of STIM1 and Orai to ER-PM contact sites 50 (Figure 2). Additionally, RASSF4 (Ras association domain-containing protein 4) 51, RPGR (retinitis pigmentosa GTPase regulator) and its interacting partners 52, and junctophilin subtypes 53 also exert positive effects on the formation of ER-PM contact sites. There are also inhibitory proteins, such as TMEM178 (transmembrane protein 178) 54, σ1Rs (sigma 1 receptors) 55, and the MT plus end-binding protein EB1 56, that downregulate SOCE. These proteins bind to STIM1, thus impeding the redistribution of STIM1 and preventing the excess activation of SOCE and overload of the ER with Ca^2+^.

There are numerous other lipid transporters with different lipid-binding domains that mediate lipid exchange between the ER and the PM to maintain lipid homeostasis and signal transduction. For instance, TMEM24 (transmembrane protein 24) is a phospholipid transfer protein that localizes at the ER and is highly expressed in the brain. The phosphorylation modification of C-terminal region in TMEM24 dominates its localization. The SMP domain of TMEM24, which binds preferentially to PI, mediates delivery of PI from the ER to the PM to replenish the pool of PtdIns(4,5)P_2_. Overexpression of TMEM24 in the neuronal cell body or dendrites significantly increases the length and the number of ER-PM contact sites that have colocalized with Kv2 in resting cells, while under the condition of increased cytosolic Ca^2+^, the C-terminal region of TMEM24 can be phosphorylated (Figure 2). This drives the dissociation of TMEM24 from the PM, which further influences the trafficking of PI 57, 58. Thus, TMEM24 has the ability to balance Ca^2+^ and lipids to promote the formation of ER-PM contact sites and trafficking of PI from the ER to the PM, supporting cellular homeostasis and further controlling neuronal excitability.

ORP5 (oxysterol-binding protein (OSBP)-related protein 5) and ORP8 are integral membrane proteins at the ER. Both of them have a pleckstrin homology (PH) domain, which tethers the protein to the PM in a PtdIns4P-dependent manner, and ORDs (OSBP-related domains) bind to PtdIns4P and PS 59, 60. Overexpression of ORP5 induces more ER-PM contact site formation, while OPR8 does not. Moreover, coexpression of ORP8 and ORP5 reduces the distribution of ORP5 at the PM. Therefore, ORP5 is the main factor promoting the formation of ER-PM contact sites. ORP8, however, has an inhibitory effect on the interaction of ORP5 and the PM in HEK293-AT1 cells because of its acidic N-terminal region 59 (Figure 2). Excess ORP5 also reduces the PtdIns4P level at the PM and increases its PS level. The exchange of PtdIns4P from the PM to the ER and of PS from the ER to the PM contributes to the regulation of the PtdIns4P level at the PM and maintenance of membrane lipid homeostasis 60.

Collectively, published studies show that the major functions of ER-PM contact sites involve Ca^2+^ and lipid exchange. For example, E-syts, ANO8, and STIMI1-Orai are associated with Ca^2+^ transfer at ER-PM contact sites. These proteins are responsible for the replenishment of Ca^2+^ stores upon Ca^2+^ release or promote the uptake of Ca^2+^ from the cytoplasm to avoid Ca^2+^ toxicity and cell death. ER Ca^2+^ homeostasis is necessary for the correct protein folding and function of chaperones. Disruption in this homeostasis leads to the accumulation of unfolding proteins to activate URP, the impairment of ER dynamics, and the induction of apoptosis 61. Therefore, promoting Ca^2+^ exchange between the ER and the PM may be a potential pathway to control the distribution of cellular Ca^2+^ to maintain cell survival in response to ER stress. On the other hand, Scs2/22, E-syts, TMEM24 and ORP5 regulate the levels of PtdIns(4,5)P_2_ and PtdIns4P at the PM. PtdIns(4,5)P_2_ at the PM is able to support the optimal conditions for ion channels at the PM, such as voltage-gated potassium channels and calcium channels. This also involves exocytosis and endocytosis. Otherwise, PtdIns(4,5)P_2_ recruits and activates several actin regulatory proteins to control cell shape, motility, and a variety of other processes with the help of PtdIns(3,4,5)P_3_ and small GTPase 62. A recent study also proposed a novel role of PtdIns(4,5)P_2_ in mediating cholesterol exchange to the PM with ORP2 (oxysterol-binding protein-related protein 2) 63. Therefore, the regulation of PtdIns(4,5)P_2_ and its precursor by ER-PM contact sites may be an important pathway to support its essential function at the PM. However, in addition to these molecules involved in Ca^2+^ and lipid exchange, other factors and their functional mechanisms at ER-PM contact sites should be investigated.

## ER-mitochondrion contact sites

**Figure 3:**
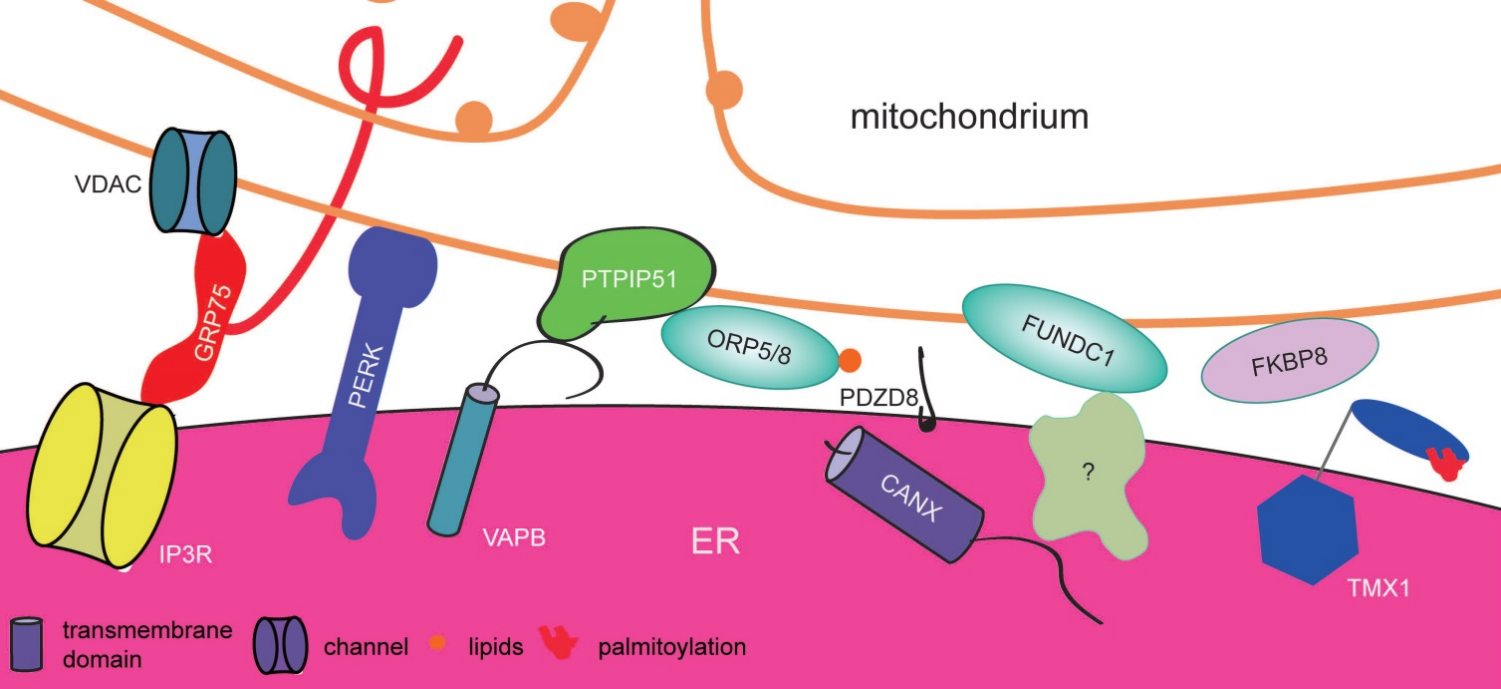
** Proteins involved in ER-mitochondrion contact sites in mammalian cells.** A variety of proteins contribute to the formation and remodeling of ER-mitochondrion contact sites. These complexes include VDAC-GRP75-IP3R, PTPIP51-VAPB and the FUNDC1-associated complex, which regulate mitochondrial dynamics. ER-resident proteins (such as PERK and TMX1) participate in the formation of ER-mitochondrion contact sites by ER stress or posttranslational modification. Whereas, FKBP8 localize at mitochondria to form MAM.

Mitochondria are the 'powerhouses' in cells, and they produce energy continuously to support essential cellular activity and for homeostasis. They take part in a large subset of primary metabolic pathways, such as fatty acid oxidation, gluconeogenesis, steroidogenesis, heme synthesis, and the urea cycle. Like the ER, mitochondria are also intracellular Ca^2+^ reservoirs. Mitochondrial dynamics, cell metabolism, and survival are regulated by Ca^2+^ or Ca^2+^-dependent enzymes, so fine-tuning of mitochondrial Ca^2+^ levels is essential to maintain their physiological function 64-68. VDACs (voltage-dependent anion channels) and MCUs (mitochondrial calcium uniporters) are two important ion channels that regulate mitochondrial Ca^2+^ uptake 69, 70 (Figure 3). It has been found that PS is mainly synthesized in the ER and converted to PE (phosphatidylethanolamine) in the mitochondria, whereas PE is delivered back to the ER to produce PC (phosphatidyl cholines). These processes require catalytic enzymes specifically localized at the ER or mitochondria 71, 72. Therefore, mechanisms are required to facilitate the rapid exchange of ions, lipids, and proteins between these two organelles. In the 1950s, Wilhelm Bernhard was the first biologist to see the ER-mitochondrion contact sites in rat liver cells under an electron microscope 73, 74. The gap between mitochondria and the smooth ER is usually approximately 10 nm, while the gap between mitochondria and the rough ER is approximately 25 nm. Approximately 2% to 5% of the mitochondrial surface is closely associated with ER membranes 75. This structure is also referred to as the mitochondria-associated ER membrane (MAM). The MAM has the ability to mediate Ca^2+^ and lipid transfer between the ER and mitochondria to regulate lipid synthesis and Ca^2+^ homeostasis, and it further influences mitochondrial dynamics (fission and fusion), autophagy, apoptosis and a broad spectrum of other essential cellular processes 76-79.

In yeast, the ERMES (ER-mitochondrion encounter structure) is a special macromolecular complex that links the ER and mitochondria. It consists of a cytosolic protein named Mdm12 (mitochondrial distribution and morphology 12), two outer mitochondrial membrane proteins Mdm34 and Mdm10, and the ER protein Mmm1 (maintenance of mitochondrial morphology 1) 71. Mmm1 and Mdm12 have SMP domains that are exposed to the cytoplasm and interact directly with each other via tail-to-tail contact, while Mdm12 binds to Mdm34 by head-to-head contact 77, 80, 81. The Mdm12-Mmm1 complex forms an extended hydrophobic tunnel through the elongated SMP domains of Mdm12 and Mmm1, which can bind glycerophospholipids such as PC, PA, PG (phosphatidylglycerol), and PS but not PE 77, 81. This specific feature of ERMESs plays a pivotal role in MAM formation by mediating lipid exchange between the ER and mitochondria via a nonvesicular pathway that does not consume energy. Interestingly, according to affinity purification of the ERMES complex and mass spectrometry, Gem1, a Miro GTPase, was found to interact with the ERMES complex. It is dispensable for ERMES assembly. Moreover, its first GTPase domain and Ca^2+^-binding domain promote its association with the ERMES assembly, and its second GTPase domain regulates its activity; however, the appropriate physiological conditions triggering Gem1 to associate with ERMES are still unknown 82. It's surprising that no ERMES homolog was known in human until the discovery of PDZD8 (PDZ domain-containing protein 8). PDZD8 is an ER-resident proteins and has a SMP domain, which may be a potential metazoan structural and functional homolog of Mmm1 in ERMES. It plays important roles in forming MAMs and mediating the Ca^2+^ crosstalk in neurons 83.

In mammals, three proteins, GRP75 (glucose-regulated protein 75), VDAC1, and IP3R, are often detected in the components of purified MAM fractions from rat livers or HeLa cells. GRP75 has a very strong mitochondrial matrix targeting sequence at the N-terminus and it is clearly shown to be localized at the mitochondrial matrix side of IP3R-VDAC complex at the MAM by the electron microscopy technique 84. Both IP3R and VDAC1 coimmunoprecipitate with GRP75 85. In HT22 cells, the absence of GRP75 restricts the formation of MAMs and attenuates the increase in mitochondrial Ca2+ induced by glutamate, preventing glutamate-induced cell death. In contrast, overexpression of GRP75 increases the number of MAMs and the susceptibility to glutamate-induced cell death 76 (Figure 3). Emerging evidence suggests that GRP75 is the scaffold protein that tethers the ER and mitochondria by interacting with IP3R and VDAC1 76, 85 and mediates Ca^2+^ exchange between these two organelles to both control mitochondrial Ca^2+^homeostasis and attenuate glutamate-induced cell death.

The formation and stability of MAMs mediated by the IP3R-GRP75-VDAC1 complex are regulated by other interacting factors of this complex. For example, the σ1R is a ligand-controlled chaperone in the MAM, and its mutation contributes to the pathogenesis of members of the ALS/FTD (amyotrophic lateral sclerosis/ frontotemporal dementia) family. Upon Ca^2+^ release, σ1R is able to stabilize channel-forming IP3R and sustain Ca^2+^ signaling from the ER into mitochondria to control cell survival in CHO cells 86-88. This reveals that the disturbance of the structure and function of MAMs may explain the mechanism of ALS caused by the aberrant type of σ1R. DISC1 (disrupted-in-schizophrenia 1) 89 interacts with IP3R and serves as a gatekeeper to regulate ER-mitochondrion Ca^2+^ transfer in response to physiological stress in cortical neurons, while the interaction of Bcl2l10 with IRBIT 90 exerts an inhibitory effect on IP3R activity to reduce Ca^2+^ transfer during apoptosis (Figure 2). PDK4 (pyruvate dehydrogenase kinase isoform 4) is able to stabilize the IP3R1-GRP75-VDAC1 complex to maintain MAM formation and suppress insulin signaling 91. Moreover, both TG2 (transglutaminase type 2) 92 and Tespa1 (thymocyte-expressed, positive selection-associated gene 1) 93 are capable of interacting with GRP75 and negatively regulating its activity, which may subsequently impair the formation of MAMs and disrupt Ca^2+^ flux.

DJ-1, encoded by the PARK7 gene, is a mitochondrial protein that responds to oxidative stress and is involved in autosomal recessive early-onset PD (Parkinson's disease) and cancer 94, 95. In DJ-1-overexpressing cells induced by cell stimulation, the colocalization of the ER and mitochondria increases, and mitochondrial Ca^2+^ transients increase, while depletion of DJ-1 results in fragmented mitochondria and reduced mitochondrial Ca^2+^ uptake. However, a high level of p53, a tumor suppressor, also decreases the number of MAMs, blocks Ca^2+^ transfer between the ER and mitochondria, and impairs mitochondrial morphology in HeLa cells. These effects are reversed by overexpression of DJ-1 96. Thus, DJ-1 may play a role opposite to that of p53 in the regulation of the formation and function of MAMs. However, both maintain the balance of mitochondrial morphology and dynamics.

Mfn2 (mitofusin-2), a GTPase dynamin-like protein of the outer mitochondrial membrane, is also considered a tether for linking the ER and mitochondria 97, 98. In addition, studies of the ubiquitination of Mfn2 by Parkin or MITOL have also indicated a role of Mfn2 in MAMs 99, 100. However, some researchers have found that the ablation of Mfn2 in MEF cells leads to an increased percentage of ER-covered mitochondria, which could be reserved by overexpression of Mfn2. Moreover, IP3-dependent Ca^2+^ release is increased in Mfn2-deficient MEF cells, and the Ca^2+^ peak in mitochondria increases 101. Collectively, these results suggest that Mfn2 appears to have an inhibitory effect on MAM formation and prevents Ca^2+^ overload and cell death, rather than acting as a tether. Thus, the function of Mfn2 in MAMs is debatable. Interestingly, the deletion of Mfn2 downregulates Aβ production by reducing γ-secretase maturation and activity 102. This means that ER-mitochondrion contact sites may control the level of Aβ (beta amyloid), which suggests a new mechanism for intervening in the pathology of AD (Alzheimer's disease).

Autophagy is a process that degrades large protein aggregates and damaged organelles 103-105. Mitophagy is a specialized form of autophagy for the degradation of mitochondria. Certain autophagy-associated proteins also participate in the formation and maintenance of MAMs. FUNDC1 (FUN14 domain-containing protein 1) is a mitophagy receptor in the OMM (outer mitochondrial membrane) 106. Its depletion disrupts the MAM and regulates mitochondrial fission and mitophagy. The mechanisms involved may widely vary in response to different cell stresses. IP3R2 is able to interact with FUNCD1 to tether the ER and mitochondria. Absence of FUNDC1 decreases the expression of IP3R2 and reduces the Ca^2+^ level in mitochondria in cardiomyocytes, while overexpression of FUNDC1 has the opposite phenotype. It is speculated that FUNDC1 regulates the activity of IP3R and the flux of Ca^2+^ between the ER and mitochondria. Alteration of the intracellular Ca^2+^ level influences the transcription of Fis1, which in turn affects mitochondrial fission by activating the transcription factor CREB (cAMP-response element binding protein) 107. However, in response to hypoxia, the depletion of FUNDC1 impairs the MAM structure, resulting in lower levels of Drp1 (dynamin-relatedprotein 1) at the MAM and more elongated mitochondria. These effects are reversed by overexpression of FUNDC1. Silencing CANX also decreases the levels of MAM-associated FUNDC1 and Drp1, thereby reducing the amount of extended mitochondria (Figure 3). This shows that CANX (calnexin) is a key protein that drives FUNDC1 to the MAM, where the former subsequently recruits Drp1 to mediate mitochondrial fission 78, 108. Because CANX and Drp1 interact with FUNDC1 via the same domain, FUNDC1 interacts with them differentially over time. It is interesting that FUNDC1 interacts indirectly with CANX at the early stage of hypoxia and then dissociates and binds directly to Drp1 78. At the later stage, hypoxia induces dephosphorylation of FUNDC1, which then interacts with LC3 (microtubule-associated proteins 1A/1B light chain 3) to promote mitophagy 106, 108. Therefore, FUNDC1 works with CANX and Drp1 at the MAM to regulate mitophagy and mitochondrial fragmentation in response to hypoxia. Thus, FUNDC1 is a novel MAM protein that mediates mitochondrial fission by activating different fission factors.

PTPIP51 (protein tyrosine phosphatase interacting protein 51) is an outer mitochondrial membrane protein that is able to induce apoptosis and control autophagy 79, 109. PTPIP51 was found to interact with VAPB at MAMs in a variety of assays in HEK293 cells 110, 111. Loss of VAPB or PTPIP51 decreases the number of MAMs, raises the Ca^2+^ level in the cytosol and delays mitochondrial Ca^2+^ uptake, while overexpression of VAPB prompts the formation of MAMs and further induces the interaction of VDAC and IP3R 79. This demonstrates that VAPB and PTPIP51 are able to tether the ER and mitochondrion to regulate Ca^2+^ exchange between these two organelles (Figure 3). Interestingly, ORP5/8 also interacts with PTPIP51 via their functional ORD domains and is targeted to the MAM rather than ER-PM contact sites in HeLa cells, which perhaps promotes PS transport to the mitochondria and regulates mitochondrial morphology. Cytoplasmic forms of TDP-43 (TAR DNA-binding protein 43) 111 and FUS 112 accumulate in ALS patient neurons, and mutations in the genes encoding them cause this disease. Both of them can activate the GSK-3β (glycogen synthase kinase-3 beta) signaling pathway, which has been found to affect the association of VAPB and PTPIP51 to disrupt the formation of MAMs and in turn affect Ca^2+^ homeostasis.

Interestingly, several stress-responsive proteins localize at MAMs. These proteins include the mature high-mannose type of pannexin 2 113, Ero1α (ER oxidoreductin 1α) 114, and RTN1A (reticulon 1A) 115. The presence of PERK (RNA-dependent protein kinase-like ER kinase) 116 and palmitoylated TMX1 (thioredoxin-related transmembrane protein 1) at the MAM promotes the remodeling of the MAM and Ca^2+^ transfer to regulate mitochondrial apoptosis 117, 118, while mouse Stbd1 (starch binding domain-containing protein 1) positively regulates MAM formation and controls mitochondrial morphology 119. Tom70 (translocase of outer membrane 70 kDa subunit) 120 not only recruits IP3R to the MAM for Ca^2+^ transfer but also recruits Ist2 for sterol transport 121, 122. VMP1 (vacuole membrane protein 1) 123 and CAV1 (caveolin-1) 124 disrupt MAM formation and control mitochondrial dynamics, while MAM-associated PML (promyelocytic leukemia protein) 125, PINK1 (PTEN induced putative kinase 1) and BECN1 (Beclin 1) 126 control autophagosome formation. Additionally, the application of enzymes with high-affinity (split-TurboID or split-BioID) in proximity labeling also discovers a variety of novel proteins in MAMs 84, 127, such as FKBP8 (FK506-binding protein 8), which participates in MAM formation; it mediates the Ca^2+^ transport from the ER to mitochondria 84. Besides, EXD2 (exonuclease 3'-5' domain-containing protein 2), ABCD3 (ATP-binding cassette sub-family D member 3), LBR (lamin B receptor), MTFR1 (mitochondrial fission regulator 1), and FUNDC2 (FUN14 domain-containing protein 2) were also found in MAMs by these technology 127.

In conclusion, ER-mitochondrial tethers are primarily involved in Ca^2+^ transfer, as well as autophagy. The regulation of both MAM formation and MAM-associated functions is important for mitochondrial morphology and dynamics in response to different cell stresses. The concentration of Ca^2+^ in mitochondria is important for mitochondrial dynamics and cell metabolism. An appropriate level of Ca^2+^ in mitochondria is able to control oxidative phosphorylation and increase the amount of ATP generated because the activity of enzymes involved in the Krebs cycle, such as pyruvate dehydrogenase phosphatase, isocitratede hydrogenase, and oxoglutarate dehydrogenase, is regulated by Ca^2+^. However, excess Ca^2+^ in mitochondria promotes the opening of the PTP (permeability transition pore), which further facilitates the release of cytochrome and activates the apoptotic cascade reaction 128. These proteins or protein complexes (such as GRP75-VDAC-IP3R, PTPIP51-VAPB, DJ-1, Mfn2, and FUNDC1) regulate the formation of MAMs, which in turn control Ca^2+^ exchange between the ER and mitochondria to modulate the level of Ca^2+^ in mitochondria. In addition, they may play an important part in the maintenance of mitochondrial homeostasis, cell metabolism, and cell survival and support a series of energy-consuming physiological processes. Furthermore, abnormal MAMs are present in several disease models, especially those of neurodegenerative diseases. For example, MAM disruption has been observed in ALS/FTD. This is mainly because the GSK-3β signaling pathway is activated in this disease model and then impairs the interaction of PTPIP51 and VAPB 111, 112. Primary fibroblasts from PD patients have been shown to reduce MAM and abnormal Ca^2+^ transfer 99. Gene mutations of Parkin and PINK1 and aberrant accumulation of mutant a-synuclein are etiologies of PD. Their knockout leads to a low level of MAM. Furthermore, rebuilt artificial MAM in PINK1-KO drosophila restores locomotion defects 99, 129. Otherwise, a-synuclein can also be found in MAMs to regulate mitochondrial morphology. Mutant a-synuclein also results in impairment of MAMs 130. AD causes the opposite phenotype. The enhancement of MAMs can be detected in AD patients and cell models 131. Under exposure to Aβ, the expression of MAM-associated proteins is increased in primary hippocampal neurons. Aberrant cleavage of the APP (amyloid precursor protein) is the main hallmark of AD pathogenesis. Mutations in PS1 and PS2, components of the ɣ-secretase complex involved in APP processing, are major causes of familial AD. PS1, PS2, APP, and ɣ-secretase are detected at MAMs. The enzymatic activity of ɣ-secretase is reduced when MAM is disrupted. Depletion of PS1 (presenilin-1) and PS2 (presenilin-2) affects the MAM structure and MAM-dependent lipid metabolism and Ca^2+^ transfer 125, 132. Therefore, MAM mutually communicates with these proteins to support their own work. Furthermore, MAM may be involved in the pathogenesis of HSP (hereditary spastic paraplegia) 133 and WS (Wolfram syndrome) 134. On the other hand, upregulation of MAM can be observed in epithelial cells from PAH (pulmonary arterial hypertension) 135, and insulin resistance has been found to be associated with downregulation of MAM in mouse models of obesity and T2D (type 2 diabetes) 136. Therefore, the formation and function of MAM may be a downstream effector for the progression of neurodegenerative disease and metabolic disease. Correcting the function of MAM in these diseases could probably be a new therapeutic target to reverse or relieve clinical symptoms. Is there any new role of MAM in cellular metabolism? Do alterations in MAM affect the development and prognosis of diseases? MAMs may have multiple tasks in regulating different physiological processes in different pathways. More questions should be answered.

## ER-Golgi contact sites

**Figure 4:**
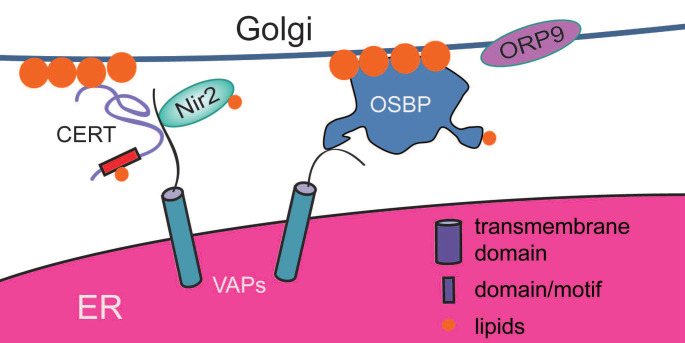
** Proteins involved in ER-Golgi contact sites in mammals.** Acting as scaffold proteins, VAPs play important roles in the organization of ER-Golgi contact sites. They are able to recruit various types of lipid transfer proteins (such as OSBP, CERT and Nir2) to ER-Golgi contact sites to promote lipid exchange between these two organelles. OSBP is a special lipid transporter with the ability to form ER-Golgi contact siteswith the help of ORP9, while CERT does not have this ability.

The Golgi apparatus is formed by a set of flattened and stacked cisternae with interconnected tubules and vesicles. The core structure is surrounded by a variety of vesicles. The Golgi is a sorting hub and modification compartment in cells 137. Normally, it receives and modifies proteins and lipids that exit from the ER. The enzymes localized in the Golgi chambers participate in catalyzing a series of posttranslational modifications. Afterward, proteins and lipids are sent to their destinations (the PM, endosomes, etc.) via the vesicle-mediated secretory pathway 138. In addition, the Golgi takes part in biophysical processes to promote cell polarization, cell migration, and mitosis 139-141. Vesicular transport between the ER and the Golgi plays an important role in the communication between these two organelles 142. A structure called the ER-to-Golgi intermediate compartment (ERGIC), which is independent of ER exit sites (ERESs) and the Golgi, mediates the anterograde and retrograde transport between these two organelles 143 (Figure 4). However, in the 1970s, the tubules of the smooth ER were found to extend to the Golgi compartment in neurons 144. In rat Sertoli cells, the ER cisternae and Golgi vesicles were demonstrated to have membrane interactions 145, and since then, the concept of ER-Golgi contact sites has been proposed. At ER-Golgi contact sites, the average distance between the membranes is 12 nm, and 55% to 60% of Golgi stacks are engaged 146. However, there are relatively few studies related to ER-Golgi contact site formation, and those have mainly focused on lipid exchange.

As scaffold proteins, VAPs play important roles in the organization of ER-Golgi contact sites. In VAP-depleted cells, the distribution of the Golgi is significantly distant from the perinuclear region, and ER-Golgi contact sites induced by 25OH are disrupted. Additionally, when VAPs are depleted, various types of lipid transfer proteins, such as OSBP (oxysterol-binding protein), CERT (ceramide transfer protein), and Nir2, lose their ability to localize at the Golgi, and the levels of PtdIns4P, sphingomyelin and diacylglycerol decrease 147, 148. Therefore, VAPs play important roles in the organization of ER-Golgi contact sites and are responsible for the recruitment of OSBP, CERT (ceramide transfer), and Nir2 (PI/PC-transfer) to the Golgi, which may further influence the lipid composition of the Golgi (Figure 4).

OSBP1 is a lipid transporter that regulates lipid metabolism. OSBP1 plays important roles in tethering the ER and the Golgi by virtue of its FFAT motif and PH domain to bind VAPs on the ER and PtdIns4P on the Golgi, respectively. Under treatment with 25OH, OSBP1 translocates to the perinuclear region where VAPs also accumulate, and extensive ER-Golgi contact sites are have in turn been observed 146. However, it is surprising to find that single depletion of either OSBP1 or ORP9 (oxysterol-binding protein-related protein 9) has no effect on ER-Golgi contact sites. Only combined depletion of OSBP1 and ORP9 dramatically influences the stability of ER-Golgi contact sites. When reducing the lipid exchange activity of OSBP1 by itraconazole in ORP9-KO cells, there is still little change in ER-Golgi contact sites 146. Therefore, the cooperation of OSBP1 and ORP9 maintains ER-Golgi contact sites, but this action is independent of the lipid transfer activity of OSBP1, although the ORD domain of OSBP1 plays an important role in lipid exchange at ER-Golgi contact sites. OSBP requires 4 steps to mediate lipid exchange 149: membrane tethering, sterol transfer, PtdIns4P transfer, and PtdIns4P hydrolysis. The PH domain and FFAT domain connect the ER and the Golgi, while its ORD domain mediates sterol transfer from the ER to the Golgi and returns a PtdIns4P molecule to the ER where phosphate is hydrolyzed by Sac1. This cycle drives the transport of sterol from the ER, its site of synthesis, to the trans-Golgi, against its concentration gradient, using the energy generated from hydrolysis of the phosphate from PtdIns4P. The consumption of PtdIns4P is in turn replenished by PI4kinases to support the next round of the four-step cycle. Therefore, OSBP1 sustains ER-Golgi contact site stability with the help of ORP9 and coordinates with PI4kinases to control the lipid composition of the ER and Golgi. It is interesting that Denisa Jamecna and his colleagues found that an N-terminal region of low amino acid complexity upstream of the PH domain is able to limit the OSBP orientation and density to ER-Golgi contact sites without any influence on its FFAT motif or PH domain 150. This may be a switch to control the function of OSBP reasonably and fully in cells.

Additionally, the OSBP-related protein ORP10 146 also contributes to the establishment of ER-Golgi contact sites, but it relies on its ORD domain with the ability to mediate lipid transfer of PS.

The yeast protein Nvj2p mainly localizes in the nucleus-vacuole junction, but it is also recruited to ER-Golgi contact sites during DTT-induced ER stress. Nvj2p depletion suppresses the number of ER-Golgi contact sites induced by DTT. The SMP domain of Nvj2p binds to ceramides; overexpression of Nvj2p results in rapid ceramide transfer 151. Therefore, Nvj2p is responsible for the formation of ER-Golgi contact sites and for ceramide transport to the Golgi at these sites to prevent ceramide toxicity during ER stress in yeast. Its homolog in humans is HT008; expressing HT008 in yeast only partially restores the function mediated by Nvj2p 151. It will be fascinating to explore whether HT008 in mammalian cells can form ER-Golgi contact sites and can coordinate with CERT to mediate ceramide transport.

In summary, the majority of proteins that re involved in the formation of ER-Golgi contact sites are lipid transfer proteins. These proteins link the Golgi to the ER with the help of VAPs. Thus, the presence of VAPs is critical for the formation of ER-Golgi contact sites. The unique function of ER-Golgi contact sites is ceramide transfer. Excess cytoplasmic ceramide is harmful to cells; it induces cell stress and autophagy and arrests growth and causes apoptosis 152. Ceramide in the Golgi can be acted upon by enzymes at the Golgi (such as sphingomyelin synthase 1 (SMS1) 153 in mammals and Aur1p 151 in yeast) and converted to a different type of sphingolipid (such as sphingomyelin and glucosylceramide) 154. ER-Golgi contact site-mediated ceramide transfer is an essential nonvesicle transport pathway for promoting the delivery of ceramide from the ER to PM. In yeast, Nvj2p not only supports the formation of ER-Golgi contact sites but also mediates ceramide transfer. In mammals, the most important Nvj2p is CERT. This protein can be recruited to ER-Golgi contact sites in a VAP-dependent manner and can regulate ceramide transfer via its START domain 155. Otherwise, the secretory pathway calcium ATPase 1 (SPCA1) has been discovered on the Golgi to take up Ca^2+^, in turn classifying and exporting secretory protein 156. The mechanism by which Ca^2+^ is transferred at the ER-Golgi contact site is similar to that at other ER-associated contact sites. In addition, do ER-Golgi contact site-localized proteins help maintain Golgi morphology? Does the formation of ER-Golgi contact sites regulate the function of the Golgi and ER? Do ER-Golgi contact sites mediate the physiological processes that regulate the dynamics of cellular membranes? Many unanswered questions merit further investigation (Figure 1 and Figure 4).

## ER-endosome contact sites

**Figure 5:**
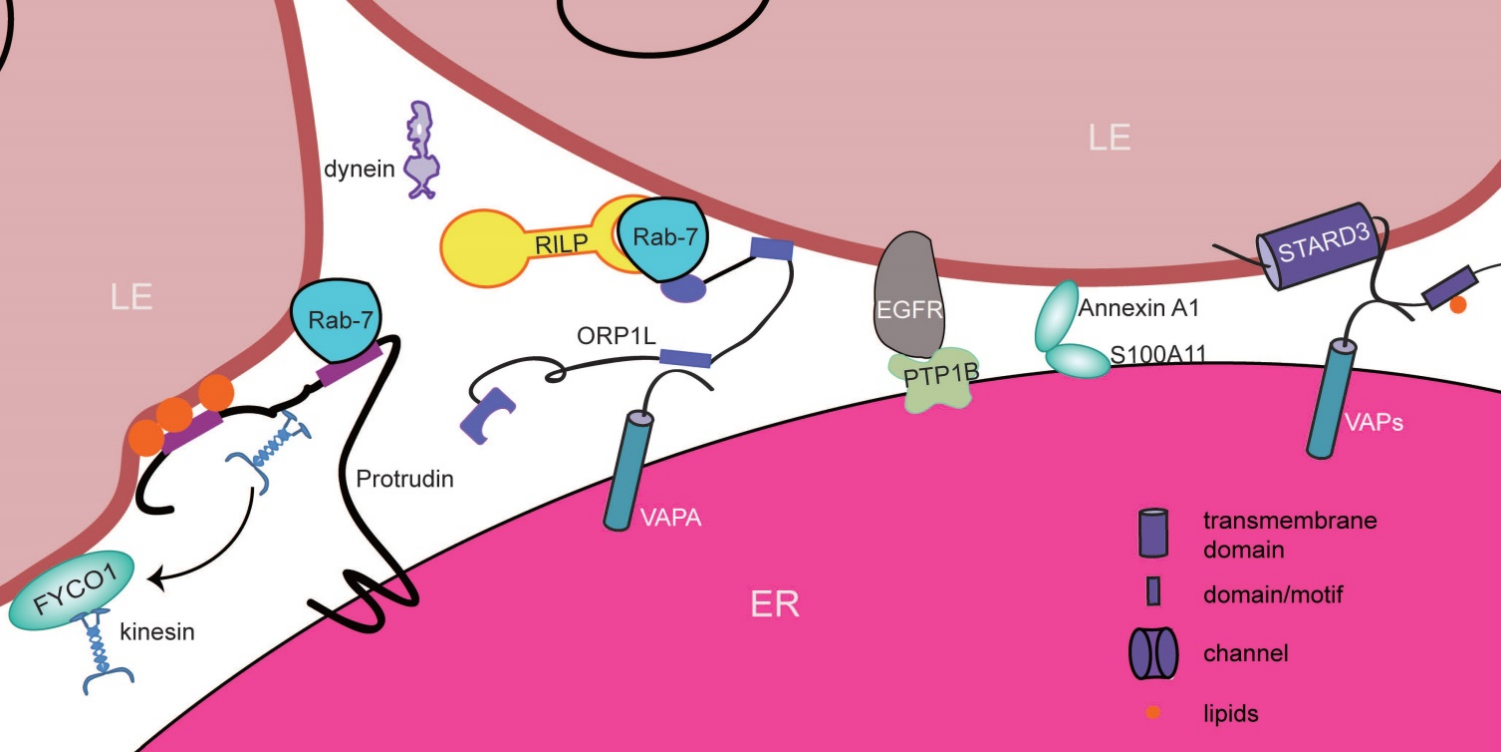
** Proteins involved in ER-LE contact sites in mammals.** Both Annexin A1-S100A11 and EGFR-PTP1B establish contacts between EGFR-positive MVBs and the ER to regulate EGF signaling. Moreover, LE motility is controlled by Rab7-associated interactions (ORP1L and protrudin). ORP1L senses the reduction in the cholesterol level and then binds to VAPA in the ER to form ER-LE contact sites. However, this process promotes the dissociation of dynein from the Rab-7-RILP complex to control the minus end-directed motility of LEs.Protrudin is able to form ER-LE contact sites with the help of Rab-7 and lipids on late endosomes. At these sites, protrudin delivers kinesin to FYCO1 to regulate plus end-directed motility of LEs. The VAP-STARD3 complex and ORP1L are responsible forsterol transfer from the ER to endosomes at these sites.

Endocytosis is a dynamic process in which many kinds of macromolecules, fluids, and particles are internalized by vesicles and vacuoles. The endocytic machinery sorts and degrades a variety of substances to maintain cellular homeostasis (Figure 5). In mammalian cells, internalized components can be delivered to early endosomes (EEs), which are marked by EEA1 157 and Rab-5A 158, 159. Early endosomes are key sorting stations that determine the fate of internalized substances. These materials then reach late endosomes (LEs), which are mainly labeled by Rab-7A 158-160. Late endosomes have the ability to fuse with lysosomes to become endolysosomes, which are the major compartments in cells that degrade proteins and facilitate the recycling of materials. When the majority of internal vesicles and limiting membranes appear in the endosomal lumen, late endosomes are referred to as multivesicular bodies (MVBs) 161. Interestingly, endosomes at different stages interact with the ER via distinct proteins or lipids, which are called ER-endosome contact sites 162-164. These interactions regulate Ca^2+^ and lipid transfer and even link to motor proteins to define the position and timing of endosome fission and to regulate endosome trafficking and maturation 165, 166.

EMC6 (ER membrane protein complex subunit 6), as its name suggests, is a transmembrane protein localized at the ER. It has been reported that Rab-5A interacts with EMC6 162. Under starvation conditions, EMC6 is upregulated, and dramatic colocalization between EEA1 and the ER occurs as a result. Likewise, Rab-5A also appears at the ER. This phenotype is reversed by the silencing of EMC6. Therefore, the Rab-5A-EMC6 complex becomes a potential candidate for the formation of contact sites between the ER and early endosomes. As mentioned above, Rab-5A is a marker of early endosomes, and it regulates autophagy by controlling the activity of PI3K 167. In EMC6-expressing cells, Rab-5A and EEA1 colocalize with the ER. This colocalization is reduced when EMC6 is lost, resulting in defective autophagy. Mutant EMC6, which still binds to Rab-5A but loses its ability to localize at the ER in U2OS cells, ultimately blocks autophagy 162. Thus, ER-localized Rab-5A has a crucial function in initiating autophagy by forming ER-EE contact sites.

EGF (epidermal growth factor) signaling, which initiates a series of signal transduction pathways, plays a significant role in the coordination of cell differentiation, survival, and motility 168-171. EGFRs (EGF receptors) are the key mediators of EGF signaling. Once EGF binds to EGFRs, the ligand/receptor complex is endocytosed. Before the fusion of endosomes with lysosomes for degradation, the complex can be delivered into MVBs where the catalytic domain of EGFRs is removed to generate the EGF signal 172. Furthermore, EGF signals can be sequestered by ILVs (intraluminal vesicles), a compartment within MVBs, to promote degradation or can be quenched via receptor dephosphorylation by protein tyrosine phosphatases, one of which is PTP1B (protein-tyrosine phosphatase 1B) 164, 172 (Figure 5). PTP1B is an ER protein that interacts with EGFRs in MVBs 173. In EGF-stimulated cells, loss of PTP1B prolongs EGFR phosphorylation and decreases the number of contact sites between EGFR-containing MVBs and the ER, whereas in PTP1B-overexpressing cells, EGFR dephosphorylation accelerates, in turn increasing the number of contact sites. Mutant PTP1B, which binds stably to EGFR proteins, promotes the formation of ER-MVB contact sites and blocks the delivery of EGFRs to lysosomes in HeLa cells 164. These data suggest that PTP1B exerts its positive effect on promoting the formation of ER-MVB contact sites with the assistance of EGFRs; in turn, EGFRs are dephosphorylated, and EGF signaling is terminated. However, it should be noted that the overexpression of PTP1B promotes ILV formation and that ILV-sequestered EGFRs accelerates the degradation of EGF signaling. Therefore, PTP1B may regulate the EGF signaling pathway two ways simultaneously.

Another protein, Annexin A1, is superior to PTP1B in mediating the formation of ER-MVB contact sites. Annexin A1 is associated with both the plasma membrane and MVBs and is considered a substrate for EGFR kinase 174. It has been previously reported that S100A11, which predominantly localizes at the ER and ER-MVB contact sites, is able to associate with Annexin A1 to form a Ca^2+^-dependent heterotetramer 174, 175. Ablation of S100A11 or Annexin A1 prevents the formation of contacts between the ER and EGFR-containing MVBs, prolongs EGF signaling, and promotes ILV formation in HeLa cells, resulting in a phenotype similar to that resulting from depletion of PTP1B. EGFR tyrosine dephosphorylation induced by overexpressing PTP1B is also suppressed by loss of Annexin A1 175, while Annexin A1 remains at the contact between the ER and MVBs in the absence of substrate-trapping PTP1B expression (Figure 5). Presumably, the Annexin A1-S100A11 complex serves as a link to attach MVBs to the ER and further promotes the interaction of PTP1B with EGFR to control EGF signaling. In addition, cholesterol is required for forming EGF-stimulated ILVs. Cholesterol is reduced in EGFR-containing MVBs in Annexin A1 null HeLa cells when cultured in lipoprotein-deficient serum media 175. Hence, Annexin A1 participates in cholesterol transport, which is supported by Annexin A1-mediated MCS formation.

However, it is surprising that ORP1L (oxysterol-binding protein-related protein 1) acts as a cholesterol transporter to support EGF-stimulated ILV formation in the absence of exogenous LDL (low density lipoprotein) 176. Under conditions in which endosomal LDL is deprived, an increasing amount of ORP1L can be recruited to ER-EGFR-containing MVBs to compensate for the sterol on the endosomes, which promotes the formation of ILV 175. Additionally, ORP1L is also considered a cholesterol sensor to mediate the formation of ER-LE contact sites and regulates the minus end-directed motility of late endosomes (Figure 5). The RAB7-RILP-p150^Glued^ complex is a key component in this process. p150^Glued^ is a subunit of the dynein motor 177. RILP (Rab-7 interacting lysosome protein) is similar to a bridge and links Rab-7 to the dynein motor 178. The level of cholesterol is able to regulate both the formation of ER-LE contact sites and the movement of LEs. At high levels of cholesterol, the number of ER-LE contact sites is decreased, and LEs are distributed near the nucleus. ORP1L plays an important role in regulating this process. Under cholesterol-rich conditions, ORP1L interacts with Rab-7. The ORD domain of ORP1L is able to promote the association of RILP and p150^Glued^, which benefits the plus end-directed motility of late endosomes (Figure 5). Under low cholesterol conditions, however, the conformation of ORP1L changes such that its FFAT motif is exposed, and the protein subsequently becomes bound to VAPA in the ER, resulting in the formation of additional ER-LE contact sites. Such binding causes p150^Glued^ to dissociate from RILP, so LEs do not move toward the perinucleus 179, 180. Therefore, ORP1L cooperates with both the Rab-7-RILP-p150^Glued^ complex and VAPA to coordinate the formation of ER-LE contact sites, which control the minus end-directed motility of LEs.

In contrast, protrudin plays an important role in endosomal plus end-directed motility. Protrudin is an ER membrane protein involved in membrane trafficking and neurite formation 181. Protrudin has a LCR (low-complexity region) and a FYVE domain, which binds Rab-7 and PtdIns(3)P (phosphatidylinositol 3-phosphate), respectively. In cells overexpressing protrudin and GTP-Rab-7, there is more protrudin on LEs accompanied by more ER-sequestrated LEs, as observed via CLEM (correlative light and electron microscopy), but GDP-bound or LCR mutant protrudin does not. However, protrudin with a mutated FYVE domain attenuates this phenomenon. Because of protein-protein interactions (protrudin-Rab7) and protein-lipid interactions (protrudin-PtdIns(3)P), the protrudin-containing ER can be recruited to late endosomes and can induce the formation of ER-LE contact sites 182. Furthermore, LEs move toward the periphery of protrudin-transfected cells, whereas siRNA-mediated depletion of protrudin results in the accumulation of LEs in the perinuclear region compared to that in control cells, which is consistent with the effects of depletion of kinesin1 and FYCO1 (FYVE and coiled-coil domain-containing protein 1) 182. FYCO1 is a motor adapter on LEs, while kinesin1 is a microtubule motor. FYCO1 interacts with the KLC2 (kinesin light chain 2) light chain and the KIF5B (kinesin-1 heavy chain) heavy chain of kinesin1 182. Via its KIF5B-binding site, protrudin also interacts with kinesin1 183. The interplay between KIF5B and FYCO1 is also reduced in cells where protrudin is depleted or where its KIF5B-binding site is mutated. However, KIF5B-FYCO1 interactions are strengthened in the presence of a high level of protrudin 182. This suggests that FYCO1 at the ER-LE contact sites promotes the interaction of KIF5B and FYCO1. It has been speculated that the formation of ER-LE contact sites is mediated by the interaction between protrudin and Rab-7/PtdIns(3)P. At the contact sites, kinesin1, with the help of protrudin, can be delivered to FYCO-associated LEs, resulting in the plus end-directed movement of FYCO1-positive LEs. LEs display slow and random motility when associated with protrudin and fast directional motility after dissociating from protrudin 182, 184. Thus, the protrudin-Rab-7/PtdIns(3)P complex is the main tether that mediates the formation of ER-LE contact sites. These sites promote the cooperation of FYCO1 and protrudin to enhance LE motility and then facilitate protrusion outgrowth and neurite formation (Figure 5).

Additionally, TMCC1 (transmembrane and coiled-coil domains protein 1) localizes in dynamic ER domains that define the position of endosome fission, while Coronin 1c, which localizes on endosomes, is required for cargo sorting 163. Depletion of either TMCC1 or Coronin 1c in Cos-7 cells decreases the number of FAM21-labeled ER-associated endosome fission buds, and the ER spends a reduced amount of time in contact with endosomal buds 163. It has been revealed that TMCC1 may coordinate with Coronin 1c to recruit dynamic ER tubules to form contact sites with endosomal buds, which contributes to ER-associated bud fission and consequent cargo sorting (Figure 5).

STARD3 (StAR-related lipid transfer protein 3) is a lipid transfer protein that coordinates sterol metabolism in late endosomes. Its MENTAL (MLN64-N-terminal) domain is responsible for its localization and lipid binding 185. STARD3NL (STARD3 N-terminal-like protein) is its associated protein. STARD3 and STARD3NL interact with VAPs and serve as scaffold proteins to promote the formation of ER-endosome contact sites, thus contributing to rapid and efficient sterol transfer from the ER to endosomes and further promoting ILV formation and altering endosomal morphology and dynamics 186 (Figure 5).

Early endosomes, late endosomes, and even endosomal buds contact the ER through the interaction of certain ER and endosomal proteins. Such contacts not only affect EGF signal transduction, lipid exchange and budding dynamics but also control endosome fission and motility. In addition to the TMCC1-Coronin 1c complex, research has shown that the spastin and ESCRT (endosomal sorting complex required for transport) protein IST1 complex localizes at the ER-endosome contact site to regulate ER-associated endosome fission. Defects in M6PR (cation-dependent mannose-6-phosphate receptor) sorting occur at the primary cortical neurons from a spastin-HSP mouse model and patient fibroblasts, disrupting lysosomal enzyme trafficking and causing abnormal lysosomal morphology. The failure of endosome fission mediated by spastin at the ER-endosome contact site is speculated to be involved in the potential pathogenesis of HSP. Additionally, protrudin and ORP1L control the plus end-directed (anterograde) and minus end-directed (retrograde) motility, respectively, of late endosomes. These components determine the fate of cargo wrapped in vesicles. Anterograde motility suggests that cargo is recycled or secreted, while retrograde movement delivers substances to the lysosome for degradation 187. It remains largely unknown whether the initially formed ER-endosome contact sites influence the transport of substances from endosomes to lysosomes at later stages or subsequently affect the formation of new ER-endosome contacts.

## ER-LD contact sites

**Figure 6:**
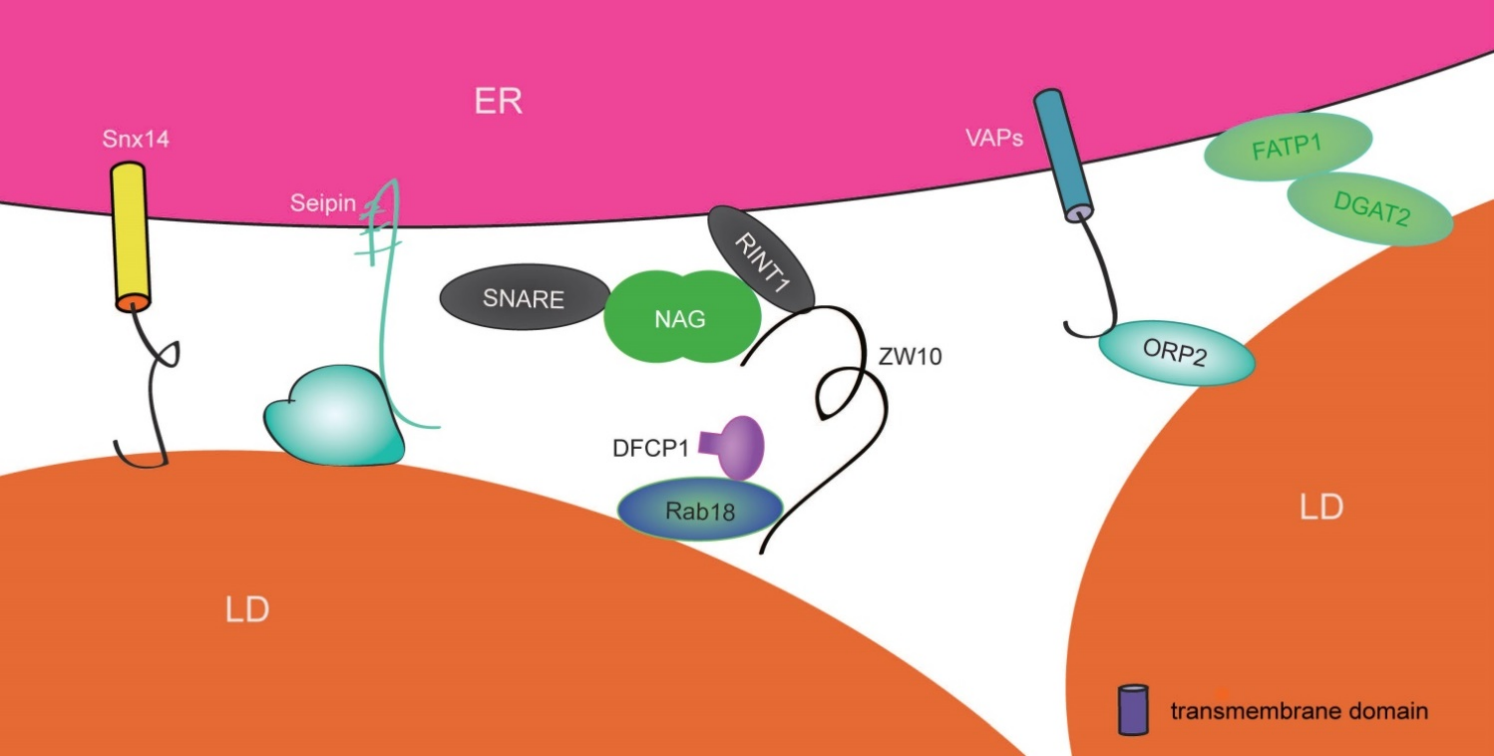
** Proteins involved in ER-LD contact sites in mammalian cells.** Rab18 interacts with the NRZ complex, Q-SNARE and DFCP to form ER-LD contact sites, thusregulating the maturation of LDs. Seipin plays important roles in organizing ER-LD contact sites and facilitating both lipid metabolism and the normal growth of LDs.However,the mechanism of Snx14 in ER-LD contact sites requires further clarification. Complexes including FATP1-DGAT2 and ORP2-VAPs shorten the distance between the ER and LDs.

Lipid droplets are lipid monolayer-enclosed organelles with abundant neutral lipids and several specific LD-related proteins. The formation of LDs relies on the ER. With the help of TAG (triacylglycerol) biosynthetic enzymes that reside at the ER, TAGs are generated and accumulate in the intermembrane space of the ER (Figure 6). A change in membrane curvature subsequently leads to the protrusion (defined as lensing) and budding of the ER membrane, which forms the nascent LD. Following TAG synthesis in either the ER or in the LD, or upon LD-LD fusion, nascent LDs keep growing and develop into mature LDs. Last, LDs can be degraded by cytosolic lipases, autophagy, or lipophagy in response to different conditions 188. It is widely known that LDs are responsible for lipid storage, but in fact, they can perform several other tasks. They take up excess fatty acids, alleviate lipotoxicity in response to cellular stress, and support the generation of energy. LDs directly contact the ER in two situations: the formation of nascent LDs from the ER and the formation of ER-LD contact sites. ER-LD contact sites facilitate the transport of enzymes, proteins, and neutral lipids, which are mainly required for maintaining the normal growth of LDs and the morphology of LDs and the ER.

Mdm1 (mitochondrial distribution and morphology protein 1) is an ER-localized protein in yeast and acts as an NVJ (ER-vacuole junction) tether. It contains an IMD (N-terminal integral membrane domain), which binds to the ER, and a PX (C-terminal phosphatidylinositol 3-phosphate-binding Phox) domain associated with the vacuole 189, 190. Interestingly, Mdm1 proteins mainly gather around NVJ at the perinuclear region and have been shown to colocalize with LDs, as observed via SIM (structure illumination microscopy). Excess Mdm1 not only promotes NVJ expansion but also increases the number of NVJ-associated LDs 191. Mdm1 probably has a potential role in regulating LD biogenesis. Replacing the vacuole-binding C-terminal region with the PM-binding region from Ist2 enables Mdm1 to interact with the PM rather than the vacuole, leading to localization of LDs at close proximity with the PM and the wrapping of ER tubules around cortical LDs, which colocalize with seipin. Therefore, Mdm1 may form and define the ER-LD contact site. Moreover, Mdm1 also includes a PXA (PX-associated) domain that targets LDs and reversibly binds to FAs (fatty acid) in vitro. The PXA domain cooperates with IMD to recruit the FA-activating enzyme Faa1 to LD buds, thus generating and transporting a high local concentration of activated FAs to LDs 189, 190. Such a process is able to provide sufficient materials to support the biogenesis and maintenance of LDs. Otherwise, it also moderates FA toxicity and maintains ER morphology during stress by converting extra FAs and then delivering them to vacuoles via lipophagy. It is interesting that yeast Mdm1 proteins are conserved in humans; their names are SNXs (sorting nexins) and include Snx13, Snx14, Snx19, and Snx25. Abundant ER-LD contact sites are observed in cells overexpressing Snx14, whose mutations are caused by a gene associated with autosomal recessive SCAR20 (spinocerebellar ataxia 20) disease. However, the mechanisms by which Snx14 and Mdm1 link LDs to the ER are different. In Snx14, an amphipathic helixin the C-nexin domain interacts with LDs and does not link to lysosomes. In addition, Snx14 is not required to provide a platform for Faa1. However, Snx14 may recruit the activating enzyme ACSL3 (acyl-CoA synthetase 3) to LD formation sites, generating a high local concentration of activated FAs 190. This is an area that requires further study. In summary, Snx14, like Mdm1, may promote the formation of ER-LD contact sites and define the features of LDs.

Fld1 (few lipid droplet protein 1) is an ER transmembrane protein in yeast 192. It has been shown that Fld1 and Ldb16 (low dye-binding protein 16) together stabilize ER-LD contact sites 193, 194. Its homolog in humans is called seipin, which is an ER-resident lipodystrophic protein. In the absence of seipin, many relatively small LDs and few relatively large LDs can be observed 192, 195. Moreover, in seipin-knockout A431 cells, the stability and characteristic neck structure of ER-LD contact sites are abnormal 195, 196, and the increased motility of LDs is independent of the ER 195. ACSL3 is an enzyme that activates FAs and generates lysophosphatidic acid, diacylglycerol, TAG, and phospholipids, which are necessary for the growth of nascent LDs 197. Under normal conditions, ACSL3 nearly completely colocalizes with LDs, while in seipin-knockout cells, it is confined at the abnormal ER-LD contact site. According to photo bleaching and “click” chemistry experiments 195, the biogenesis of neutral lipids and cargo delivery from the ER to LDs are effective at nascent LDs from seipin ablation cells, but for those preexisting LDs, such processes were impaired. Therefore, it is reasonable that part of the seipin pool localizes at ER-LD contact sites and maintains the structure and function of the ER-LD contact site where the ER connects with nascent LDs. Seipin facilitates protein and lipid transfer from the ER to preexisting LDs, which promotes the growth and maturation of nascent LDs. The mutation of BSCL2, the gene encoding seipin, is the common reason for Berardinelli-Seip congenital lipodystrophy 2 198. In primary fibroblasts from patients, the same phenotype can be observed 195. Therefore, the abnormal ER-LD contact site may be a potential target for overcoming this disease. However, the typical feature of this disease is the lack of adipose tissue in patients, while the depletion of seipin results in the accumulation of smaller LDs. It is discussable, and more research is necessary.

Rab18 is a Rab guanosine triphosphatase in LDs 199 and mediates the growth and maturation of LDs by facilitating ER-synthesized TAG transport to LDs through an unknown pathway 200. Rab18 depletion results in abnormal LD morphology, a few supersized LDs, and numerous smaller LDs upon OA (oleic acid) treatment. The interaction between the GTP-bound form of Rab18 and the NAG-RINT1-ZW10 (NRZ) complex has been identified by pull-down and immunoprecipitation assays in TM-3 Leydig cells. ZW10 acts as a bridge to link Rab18 and NAG-RINT1 by its C-terminal region and N-terminal region, respectively 200. Q-SNARE, which localizes at the ER, has also been recognized to interact with the NRZ complex 201 (Figure 6). The resulting phenotype occurs in the absence of the NRZ complex or SNARE is similar to that in Rab18-deficientcells, but the phenotype fails to be rescued by overexpression of Rab18 200. It is reasonable to speculate that the NRZ complex and its interactor Q-SNARE may be downstream effectors of GTP-bound Rab18 to control LD growth. Interestingly, the components of the NRZ complex and Q-SNARE can also be detected in the LD fraction of Leydig cells but are not detected in Rab18-depleted cells. An increased amount of LDs were associated with the ER when Rab18 was overexpressed, and the number of ER-LD contact sites significantly decreased when Rab18 was silenced 200.These findings suggest that Rab18 is likely to recruit the NRZ complex and its ER-SNARE interactors to LDs to promote the formation of ER-LD contact sites, which may mediate lipid transfer between the ER and LDs to facilitate the growth of nascent LDs into mature LDs. In addition, DFCP1 (double FYVE-containing protein 1), a protein involved in membrane trafficking and early events in the generation of autophagosomes 202, 203, interacts with Rab18 cells treated with OA. Deletion of DFCP1 results in a phenotype similar to that resulting from Rab18 knockdown. Overexpression of DFCP1 results in an increased number of ER-LD contact sites, which could be inhibited byRab18 deficiency 203, 204. Hence, DFCP1 associates with Rab18 to promote ER-LD contact sites, which in turn affects the morphology, diameter, and maturation of LDs under nutrient-rich conditions.

Additionally, both FATP1 (fatty acid transport protein 1) and DGAT2 (diacylglycerol O-acyltransferase 2) 205 contribute to LD expansion. Surprisingly, based on the results from co-immunoprecipitation assays, FATP1 was found to interact with DGAT2 in HEK293T cells. This physical interaction drives them to ER-LD contact sites, where they coordinately promote LD expansion. As a member of the OSBP family, ORP2 206 is able to bind LDs. It contains an FFAT-binding motif that binds to VAPs to shorten the distance between the ER and LDs, thus balancing TAG synthesis and TAG hydrolysis. VMP1 207, an ER-resident multiple transmembrane protein, exerts a negative regulatory effect on the formation of ER-LD contact sites, thus preventing the biogenesis of aberrant LDs in a SERCA2- and CaM-dependent pathway (Figure 6).

In summary, different proteins that mediate the formation of ER-LD contact sites have diverse functions. For example, the main function of Mdm1 is to control LD biogenesis and define LD budding sites, while Rab18 and seipin tend to work on LD growth and maturation. They regulate LD shape, size, and localization and subsequently can maintain the normal function of LDs in response to cell stress. What function occurs in response to different energetic stresses? Whether and how does it regulate the dynamics and function of the ER? How does it regulate the occurrence and development of certain diseases? These mysteries of ER-LD contact sites have not been solves.

## ER-ribosome contact sites

Of note, there's one form of contact site that is relatively less studied. Some parts of the ER are covered with ribosomes on the surface, which is called the rough endoplasmic reticulum (RER), and is commonly found in secretory cells 208. The SR (signal recognition particle receptor)-SRP (signal recognition particle)-RNC (ribosome-nascent chain complex) complex plays a significant role in the recruitment of ribosomes to the ER. SRP recognizes the signal sequence from nascent peptides, and binds to the SR on the ER surface, then recruits the RNC to the ER. Subsequently, RNC can be transferred to Sec61β (protein transport protein Sec61 subunit beta) translocon in a GTP-dependent pathway to initiate the translation of proteins. Finally, ribosomes are released from the ER to the cytosol at the end of the translation 209, 210. It has been reported that LRRC59 (leucine-richrepeat-containing59) 209 and ribosomal protein L7 211 perform functions associated with the retention of ribosomes on the ER after the translation terminate. However, what proteins are structurally mediating the direct contacts between the ER and ribosomes remain largely unknown.

## Conclusion

The extensive tubular network of the ER penetrates the whole cell and establishes intimate relationships with multiple membranes and organelles, such as the plasma membrane, mitochondria, endosomes, and lipid droplets. Such nonvesicular transport pathways strengthen the molecular connections and delivery of signals between these organelles and further regulate a series of essential cellular processes in response to a diverse array of normal physiological stimuli and cell stresses. In this review, we have presented a large group of molecules that function at these ER-associated contact sites, which play multiple roles in different physiological and pathological conditions.

Abnormal ER-associated contact sites have been identified in various diseases. Altered MAMs have been observed in Parkinson's disease 212, Alzheimer's disease 213, and Wolfram syndrome type 2 134. Mutant VPS13C 214 in ER-endosome contact sites is responsible for Parkinson's disease, while Seipin 192 in ER-LD contact sites contributes to Berardinelli-Seip congenital lipodystrophy type 2. Abnormal formation of contact sites may be a new disease mechanism, and these sites may be potential targets for therapeutic strategies.

Recently, two proximity labeling tools (Contact-ID and Split-Turbo ID) were developed and over 100 proteins were identified at MAMs in live cells 84, 127. Contact-ID, a split-BioID method, which specifically biotinylates the proteins localized at MAMs; they are subsequently identified by mass spectrometry. This method can be also utilized to profile the local proteome at any organelle-membrane contact site in live cells, thus providing a very powerful tool to study contact proteome. Split-Turbo ID, a promiscuous biotinylating enzyme, split into two inactive fragments, which can then be brought together by organelle contact through protein-protein interaction or by drugs to reconstitute its enzymatic activity. It thus provides a valuable tool for conditional or higher-specificity proximity labeling method. In the future, combining high-resolution techniques (especially electron microscope) and the proximity-based labeling methods with proteomics will provide a convenient way to discover novel molecules involved in ER-associated contact sites that mediate biological processes. Clearer working models of vesicular and nonvesicular transport, synergism or antagonism of different molecules in the same contact sites, and the different diseases related to contact sites await further exploration.

## Figures and Tables

**Table 1:** Potential tether proteins for the formation of ER-mediated membrane contacts in mammalian cells.

Contact sites	Tether Protein	Functions
**ER-PM contact sites**	E-syts	Homolog of tricalbins, Ca^2+^ transport and lipid transfer
	ANO8	Homolog of Ist2, Ca^2+^ transport
	Kv2-VAPA complex	PI transfer
	STIM1-Orai complex	Ca^2+^ transport
	TMEM24	PI transfer from the ER to the PM
	ORP5	PtdIns4P transfer from the PM to the ER and PS transfer from the ER to the PM
**ER-Mitochondria contact sites**	PDZD8	Homolog of Mmm1 in ERMES, Ca^2+^ transport
	IP3R-GRP75-VDAC1 complex	Ca^2+^ transport
	DJ-1	Maintainance of mitochondrial morphology and dynamics
	FUNDC1	Regulation of mitochondrial fission and mitophagy
	PTPIP51-VAPB	Ca^2+^ transport
	PERK, TMX1	Ca^2+^ transport, maintainance of mitochondrial morphology
	FKBP8	Ca^2+^ transport
**ER-Golgi contact sites**	OSBP-VAPs, ORP9	Sterol transfer from the ER to the Golgi and PtdIns4P transfer from the Golgi to the ER
	ORP10	PS transfer
	HT008	Homolog of Nvj2p, ceramide transport
**ER-endosome contact sites**	Rab-5A-EMC6 complex	Autophagy activation
	PTP1B-EGFR complex	Regulation of EGF signaling
	Annexin A1-S100A11 complex	Regulation of EGF signaling and cholesterol transfer
	ORP1L-VAPA	Regulation of minus-end-directed movement of endosomes
	protrudin-Rab-7/PtdIns(3)P	Regulation of plus-end-directed movement of endosomes
	TMCC1and Coronin1c	ER-associated bud fission and cargo sorting
	STARD3-VAPs	Sterol transfer from the ER to endosomes
**ER-LDs contact sites**	Snx14	Homolog of Mdm1, LD biogenesis.
	Seipin	Homolog of Fld1, protein and lipid transfer, growth and maturation of nascent LDs
	Rab18	Lipid transfer, LD maturation
	DFCP1	LD maturation
	FATP1-DGAT2 complex	LD expansion
	ORP2-VAPs	TAG metabolism

## Abbreviations

MCSs: membrane contact sites
ER: endoplasmic reticulum
PM: plasma membrane
LD: lipid droplet
IP3R: inositol 1,4,5-trisphosphate receptors
PS: phosphatidylserine
RER: rough endoplasmic reticulum
SRP: signal recognition particle
SR: SRP receptor
RNC: ribosome-nascent chain complex
Sec61β: protein transport protein Sec61 subunit beta
LRRC59: leucine-rich repeat-containing 59
PtdIns(4,5)P2: phosphatidylinositol 4,5-bisphosphate
PtdIns4P: phosphatidylinositol 4-phosphate
VAMP: vesicle-associated membrane protein
VAP: vesicle-associated membrane protein-associated protein
MSP: major sperm protein
FFAT: diphenylalanine in an acidic tract
Ist2: increases sodium tolerance protein 2
PI: phosphatidylinositol
PA: phosphatidic acid
ANO: Anoctamin
STIM1: stromal interaction molecule 1
SERCA2: sarcoplasmic/endoplasmic reticulum calcium ATPase 2
PRC: proximal restriction and clustering
VAPA: vesicle-associated membrane protein-associated protein
SOCE: “Store-operated” Ca^2+^ entry
CRAC: Ca^2+^release-activated Ca^2+^
SAM: sterile alpha-motif domain
ERM: ezrin-radixin-moesin
SOAR: STIM1-Orai1 activating region
STMATE: STIM-activating enhancer
RASSF4: Ras association domain-containing protein 4
RPGR: retinitis pigmentosa GTPase regulator
TMEM178: transmembrane protein 178
σ1Rs: sigma 1 receptors
TMEM24: transmembrane protein 24
ORP5: oxysterol-binding protein (OSBP)-related protein 5
PH: apleckstrin homology
ORDs: OSBP-related domains
ORP2: oxysterol-binding protein-related protein 2
VDACs: voltage-dependent anion channels
MCUs: mitochondrial calcium uniporters
PE: phosphatidylethanolamine
PC: phosphatidyl cholines
ERMES: ER-mitochondrion encounter structure
MAM: mitochondria-associated ER membrane
Mdm12: mitochondrial distribution and morphology 12
Mmm1: maintenance of mitochondrial morphology 1
PG: phosphatidylglycerol
PDZD8: PDZ domain-containing protein 8
GRP75: glucose-regulated protein 75
ALS/FTD: amyotrophic lateral sclerosis/ frontotemporal dementia
DISC1: disrupted-in-schizophrenia 1
PDK4: pyruvate dehydrogenase kinase isoform 4
TG2: transglutaminase type 2
Tespa1: thymocyte-expressed, positive selection-associated gene
PD: Parkinson's disease
Mfn2: mitofusin-2
Aβ: beta amyloid
AD: Alzheimer's disease
FUNDC1: FUN14 domain-containing protein 1
OMM: outer mitochondrial membrane
CREB: cAMP-response element binding protein
Drp1: dynamin-related protein 1
CANX: calnexin
LC3: microtubule-associated proteins 1A/1B light chain 3
PTPIP51: protein tyrosine phosphatase interacting protein 51
TDP-43: TAR DNA-binding protein 43
GSK-3β: glycogen synthase kinase-3 beta
Ero1α: ER oxidoreductin 1α
RTN1A: reticulon 1A
PERK: RNA-dependent protein kinase-like ER kinase
TMX1: thioredoxin-related transmembrane protein 1
Stbd1: starch binding domain-containing protein 1
Tom70: translocase of outer membrane 70 kDa subunit
VMP1: vacuole membrane protein 1
CAV1: caveolin-1
PML: promyelocytic leukemia protein
PINK1: PTEN induced putative kinase 1
BECN1: Beclin 1
EXD2: exonuclease 3'-5' domain-containing protein 2
ABCD3: ATP-binding cassette sub-family D member 3
LBR: lamin B receptor
MTFR1: mitochondrial fission regulator 1
FUNDC2: FUN14 domain-containing protein 2
FKBP8: FK506-binding protein 8
PTP: permeability transition pore
APP: amyloid precursor protein
PS1: presenilin-1
PS2: presenilin-2
HSP: hereditary spastic paraplegia
WS: Wolfram syndrome
PAH: pulmonary arterial hypertension
T2D: type 2 diabetes
ERGIC: ER-to-Golgi intermediate compartment
ERES: ER exit site
OSBP: oxysterol-binding protein
CERT: ceramide transfer protein
ORP9: oxysterol-binding protein-related protein 9
EE: early endosome
LE: late endosome
MVB: multivesicular body
EMC6: ER membrane protein complex subunit 6
EGF: Epidermal growth factor
EGFR: EGF receptor
ILV: intraluminal vesicle
PTP1B: protein-tyrosine phosphatase 1B
ORP1L: oxysterol-binding protein-related protein 1
LDL: low density lipoprotein
RILP: Rab-7 interacting lysosome protein
LCR: low-complexity region
PtdIns(3)P: phosphatidylinositol 3-phosphate
CLEM: correlative light and electron microscopy
FYCO1: FYVE and coiled-coil domain-containing protein 1
KLC: kinesin light chain 2
KIF5B: kinesin-1 heavy chain
TMCC1: transmembrane and coiled-coil domains protein 1
STARD3: StAR-related lipid transfer protein 3
MENTA: MLN64-N-terminal
STARD3NL: STARD3 N-terminal-like protein
ESCRT: endosomal sorting complex required for transport
M6PR: cation-dependent mannose-6-phosphate receptor
TAG: triacylglycerol
Mdm1: mitochondrial distribution and morphology protein 1
IMD: N-terminal integral membrane domain
PX: C-terminal phosphatidylinositol 3-phosphate - binding Phox
SIM: structure illumination microscopy
NVJ: ER-vacuole junction
SMS1: sphingomyelin synthase 1
SPCA1: secretory pathway calcium ATPase 1
PXA: PX-associated
FA: fatty acid
SNX: sorting nexin
ACSL3: acyl-CoA synthetase 3
SCAR20: spinocerebellar ataxia 20
Fld1: few lipid droplet protein 1
Ldb16: low dye-binding protein 16
OA: oleic acid
DFCP1: double FYVE-containing protein 1
FATP1: fatty acid transport protein 1
DGAT2: diacylglycerol O-acyltransferase 2

## References

[1] Westrate LM, Lee JE, Prinz WA, Voeltz GK (2015). Form follows function: The importance of endoplasmic reticulum shape. Annu Rev Biochem.

[2] Zhang H, Hu J (2016). Shaping the endoplasmic reticulum into a social network. Trends Cell Biol.

[3] Samtleben S, Jaepel J, Fecher C, Andreska T, Rehberg M, Blum R Direct imaging of er calcium with targeted-esterase induced dye loading (ted). J Vis Exp. 2013: e50317.

[4] Clapham DE (2007). Calcium signaling. Cell.

[5] Fagone P, Jackowski S (2009). Membrane phospholipid synthesis and endoplasmic reticulum function. J Lipid Res.

[6] Baumann O, Walz B (2001). Endoplasmic reticulum of animal cells and its organization into structural and functional domains. Int Rev Cytol.

[7] Zhang Z, Gao W, Zhou L, Chen Y, Qin SY, Zhang L (2019). Repurposing brigatinib for the treatment of colorectal cancer based on inhibition of er-phagy. Theranostics.

[8] Tan JH, Cao RC, Zhou L, Zhou ZT, Chen HJ, Xu J (2020). Atf6 aggravates acinar cell apoptosis and injury by regulating p53/aifm2 transcription in severe acute pancreatitis. Theranostics.

[9] Elbaz Y, Schuldiner M (2011). Staying in touch: The molecular era of organelle contact sites. Trends Biochem Sci.

[10] Giacomello M, Pyakurel A, Glytsou C, Scorrano L (2020). The cell biology of mitochondrial membrane dynamics. Nat Rev Mol Cell Biol.

[11] Zhao YG, Zhang H (2018). Formation and maturation of autophagosomes in higher eukaryotes: A social network. Curr Opin Cell Biol.

[12] Hugenroth M, Bohnert M (2020). Come a little bit closer! Lipid droplet-er contact sites are getting crowded. Biochim Biophys Acta Mol Cell Res.

[13] Prinz WA, Toulmay A, Balla T (2020). The functional universe of membrane contact sites. Nat Rev Mol Cell Biol.

[14] Sargsyan Y, Thoms S (2020). Staying in healthy contact: How peroxisomes interact with other cell organelles. Trends Mol Med.

[15] Masone MC, Morra V, Venditti R (2019). Illuminating the membrane contact sites between the endoplasmic reticulum and the trans-golgi network. FEBS Lett.

[16] Azimi I, Monteith GR (2016). Plasma membrane ion channels and epithelial to mesenchymal transition in cancer cells. Endocr Relat Cancer.

[17] Hammond GRV, Balla T (2015). Polyphosphoinositide binding domains: Key to inositol lipid biology. Biochim Biophys Acta Mol Cell Biol Lipids.

[18] Franzini-Armstrong C, Jorgensen AO (1994). Structure and development of e-c coupling units in skeletal muscle. Annu Rev Physiol.

[19] Rosenbluth J (1962). Subsurface cisterns and their relationship to the neuronal plasma membrane. J Cell Biol.

[20] Chang C-L, Chen Y-J, Liou J (2017). Er-plasma membrane junctions: Why and how do we study them?. Biochim Biophys Acta Mol Cell Res.

[21] Manford AG, Stefan CJ, Yuan HL, Macgurn JA, Emr SD (2012). Er-to-plasma membrane tethering proteins regulate cell signaling and er morphology. Dev Cell.

[22] Kagiwada S, Hashimoto M (2007). The yeast vap homolog scs2p has a phosphoinositide-binding ability that is correlated with its activity. Biochem Biophys Res Commun.

[23] Loewen CJ, Levine TP (2005). A highly conserved binding site in vesicle-associated membrane protein-associated protein (vap) for the ffat motif of lipid-binding proteins. J Biol Chem.

[24] Stefan CJ, Manford AG, Baird D, Yamada-Hanff J, Mao Y, Emr SD (2011). Osh proteins regulate phosphoinositide metabolism at er-plasma membrane contact sites. Cell.

[25] Maass K, Fischer MA, Seiler M, Temmerman K, Nickel W, Seedorf M (2009). A signal comprising a basic cluster and an amphipathic alpha-helix interacts with lipids and is required for the transport of ist2 to the yeast cortical er. J Cell Sci.

[26] Saheki Y, De Camilli P (2017). The extended-synaptotagmins. Biochim Biophys Acta Mol Cell Res.

[27] Wolf W, Kilic A, Schrul B, Lorenz H, Schwappach B, Seedorf M (2012). Yeast ist2 recruits the endoplasmic reticulum to the plasma membrane and creates a ribosome-free membrane microcompartment. PLoS One.

[28] Collado J, Kalemanov M, Campelo F, Bourgoint C, Thomas F, Loewith R (2019). Tricalbin-mediated contact sites control er curvature to maintain plasma membrane integrity. Dev Cell.

[29] Giordano F, Saheki Y, Idevall-Hagren O, Colombo SF, Pirruccello M, Milosevic I (2013). Pi(4,5)p(2)-dependent and ca(2+)-regulated er-pm interactions mediated by the extended synaptotagmins. Cell.

[30] Chang CL, Hsieh TS, Yang TT, Rothberg KG, Azizoglu DB, Volk E (2013). Feedback regulation of receptor-induced ca2+ signaling mediated by e-syt1 and nir2 at endoplasmic reticulum-plasma membrane junctions. Cell Rep.

[31] Kang F, Zhou M, Huang X, Fan J, Wei L, Boulanger J (2019). E-syt1 re-arranges stim1 clusters to stabilize ring-shaped er-pm contact sites and accelerate ca(2+) store replenishment. Sci Rep.

[32] Toulmay A, Prinz WA (2012). A conserved membrane-binding domain targets proteins to organelle contact sites. J Cell Sci.

[33] Schauder CM, Wu X, Saheki Y, Narayanaswamy P, Torta F, Wenk MR (2014). Structure of a lipid-bound extended synaptotagmin indicates a role in lipid transfer. Nature.

[34] Jha A, Chung WY, Vachel L, Maleth J, Lake S, Zhang G (2019). Anoctamin 8 tethers endoplasmic reticulum and plasma membrane for assembly of ca(2+) signaling complexes at the er/pm compartment. EMBO J.

[35] Kirmiz M, Palacio S, Thapa P, King AN, Sack JT, Trimmer JS (2018). Remodeling neuronal er-pm junctions is a conserved nonconducting function of kv2 plasma membrane ion channels. Mol Biol Cell.

[36] Kirmiz M, Vierra NC, Palacio S, Trimmer JS (2018). Identification of vapa and vapb as kv2 channel-interacting proteins defining endoplasmic reticulum-plasma membrane junctions in mammalian brain neurons. J Neurosci.

[37] Johnson B, Leek AN, Solé L, Maverick EE, Levine TP, Tamkun MM (2018). Kv2 potassium channels form endoplasmic reticulum/plasma membrane junctions via interaction with vapa and vapb. Proc Natl Acad Sci U S A.

[38] Deutsch E, Weigel AV, Akin EJ, Fox P, Hansen G, Haberkorn CJ (2012). Kv2.1 cell surface clusters are insertion platforms for ion channel delivery to the plasma membrane. Mol Biol Cell.

[39] Feinshreiber L, Singer-Lahat D, Friedrich R, Matti U, Sheinin A, Yizhar O (2010). Non-conducting function of the kv2.1 channel enables it to recruit vesicles for release in neuroendocrine and nerve cells. J Cell Sci.

[40] Kirmiz M, Gillies TE, Dickson EJ, Trimmer JS (2019). Neuronal er-plasma membrane junctions organized by kv2-vap pairing recruit nir proteins and affect phosphoinositide homeostasis. J Biol Chem.

[41] Liou J, Fivaz M, Inoue T, Meyer T (2007). Live-cell imaging reveals sequential oligomerization and local plasma membrane targeting of stromal interaction molecule 1 after ca2+ store depletion. Proc Natl Acad Sci U S A.

[42] Butorac C, Muik M, Derler I, Stadlbauer M, Lunz V, Krizova A (2019). A novel stim1-orai1 gating interface essential for crac channel activation. Cell Calcium.

[43] Chang C-L, Hsieh T-S, Yang TT, Rothberg Karen G, Azizoglu DB, Volk E (2013). Feedback regulation of receptor-induced ca2+ signaling mediated by e-syt1 and nir2 at endoplasmic reticulum-plasma membrane junctions. Cell Rep.

[44] Hoth M, Penner R (1992). Depletion of intracellular calcium stores activates a calcium current in mast cells. Nature.

[45] Yuan JP, Zeng W, Dorwart MR, Choi YJ, Worley PF, Muallem S (2009). Soar and the polybasic stim1 domains gate and regulate orai channels. Nat Cell Biol.

[46] Zhou Y, Nwokonko RM, Cai X, Loktionova NA, Abdulqadir R, Xin P (2018). Cross-linking of orai1 channels by stim proteins. Proc Natl Acad Sci U S A.

[47] Wu MM, Buchanan J, Luik RM, Lewis RS (2006). Ca2+ store depletion causes stim1 to accumulate in er regions closely associated with the plasma membrane. J Cell Biol.

[48] Jing J, He L, Sun A, Quintana A, Ding Y, Ma G (2015). Proteomic mapping of er-pm junctions identifies stimate as a regulator of ca(2)(+) influx. Nat Cell Biol.

[49] Quintana A, Rajanikanth V, Farber-Katz S, Gudlur A, Zhang C, Jing J (2015). Tmem110 regulates the maintenance and remodeling of mammalian er-plasma membrane junctions competent for stim-orai signaling. Proc Natl Acad Sci U S A.

[50] Sharma S, Quintana A, Findlay GM, Mettlen M, Baust B, Jain M (2013). An sirna screen for nfat activation identifies septins as coordinators of store-operated ca2+ entry. Nature.

[51] Chen YJ, Chang CL, Lee WR, Liou J (2017). Rassf4 controls soce and er-pm junctions through regulation of pi(4,5)p2. J Cell Biol.

[52] Patnaik SR, Zhang X, Biswas L, Akhtar S, Zhou X, Kusuluri DK (2018). Rpgr protein complex regulates proteasome activity and mediates store-operated calcium entry. Oncotarget.

[53] Takeshima H, Hoshijima M, Song LS (2015). Ca(2)(+) microdomains organized by junctophilins. Cell Calcium.

[54] Yang ZF, Yan H, Dai WT, Jing J, Yang YH, Mahajan S (2019). Tmem178 negatively regulates store-operated calcium entry in myeloid cells via association with stim1. J Autoimmun.

[55] Srivats S, Balasuriya D, Pasche M, Vistal G, Edwardson JM, Taylor CW (2016). Sigma1 receptors inhibit store-operated ca2+ entry by attenuating coupling of stim1 to orai1. J Cell Biol.

[56] Chang CL, Chen YJ, Quintanilla CG, Hsieh TS, Liou J (2018). Eb1 binding restricts stim1 translocation to er-pm junctions and regulates store-operated ca(2+) entry. J Cell Biol.

[57] Sun EW, Guillen-Samander A, Bian X, Wu Y, Cai Y, Messa M (2019). Lipid transporter tmem24/c2cd2l is a ca(2+)-regulated component of er-plasma membrane contacts in mammalian neurons. Proc Natl Acad Sci U S A.

[58] Lees JA, Messa M, Sun EW, Wheeler H, Torta F, Wenk MR (2017). Lipid transport by tmem24 at er-plasma membrane contacts regulates pulsatile insulin secretion. Science.

[59] Sohn M, Korzeniowski M, Zewe JP, Wills RC, Hammond GRV, Humpolickova J (2018). Pi(4,5)p2 controls plasma membrane pi4p and ps levels via orp5/8 recruitment to er-pm contact sites. J Cell Biol.

[60] Chung J, Torta F, Masai K, Lucast L, Czapla H, Tanner LB (2015). Intracellular transport. Pi4p/phosphatidylserine countertransport at orp5- and orp8-mediated er-plasma membrane contacts. Science.

[61] Krebs J, Agellon LB, Michalak M (2015). Ca2+ homeostasis and endoplasmic reticulum (er) stress: An integrated view of calcium signaling. Biochem Biophys Res Commun.

[62] Di Paolo G, De Camilli P (2006). Phosphoinositides in cell regulation and membrane dynamics. Nature.

[63] Wang H, Ma QL, Qi YF, Dong JQ, Du XM, Rae J (2019). Orp2 delivers cholesterol to the plasma membrane in exchange for phosphatidylinositol 4, 5-bisphosphate (pi(4,5)p-2). Mol Cell.

[64] Zavodnik IB (2016). Mitochondria, calcium homeostasis and calcium signaling. Biomed Khim.

[65] Orrenius S, Gogvadze V, Zhivotovsky B (2015). Calcium and mitochondria in the regulation of cell death. Biochem Biophys Res Commun.

[66] McCormack JG, Denton RM (1990). The role of mitochondrial ca2+ transport and matrix ca2+ in signal transduction in mammalian tissues. Biochim Biophys Acta.

[67] Sun C, Liu XX, Wang B, Wang ZH, Liu Y, Di CX (2019). Endocytosis-mediated mitochondrial transplantation: Transferring normal human astrocytic mitochondria into glioma cells rescues aerobic respiration and enhances radiosensitivity. Theranostics.

[68] Sharma A, Liaw K, Sharma R, Zhang Z, Kannan S, Kannan RM (2018). Targeting mitochondrial dysfunction and oxidative stress in activated microglia using dendrimer-based therapeutics. Theranostics.

[69] Baughman JM, Perocchi F, Girgis HS, Plovanich M, Belcher-Timme CA, Sancak Y (2011). Integrative genomics identifies mcu as an essential component of the mitochondrial calcium uniporter. Nature.

[70] Okada SF, O'Neal WK, Huang P, Nicholas RA, Ostrowski LE, Craigen WJ (2004). Voltage-dependent anion channel-1 (vdac-1) contributes to atp release and cell volume regulation in murine cells. J Gen Physiol.

[71] Kornmann B, Currie E, Collins SR, Schuldiner M, Nunnari J, Weissman JS (2009). An er-mitochondria tethering complex revealed by a synthetic biology screen. Science.

[72] Flis VV, Daum G (2013). Lipid transport between the endoplasmic reticulum and mitochondria. Cold Spring Harb Perspect Biol.

[73] Bernhard W, Haguenau F, Gautier A, Oberling C (1952). Submicroscopical structure of cytoplasmic basophils in the liver, pancreas and salivary gland; study of ultrafine slices by electron microscope. Z Zellforsch Mikrosk Anat.

[74] Bernhard W, Rouiller C (1956). Close topographical relationship between mitochondria and ergastoplasm of liver cells in a definite phase of cellular activity. J Biophys Biochem Cytol.

[75] Csordas G, Renken C, Varnai P, Walter L, Weaver D, Buttle KF (2006). Structural and functional features and significance of the physical linkage between er and mitochondria. J Cell Biol.

[76] Honrath B, Metz I, Bendridi N, Rieusset J, Culmsee C, Dolga AM (2017). Glucose-regulated protein 75 determines er-mitochondrial coupling and sensitivity to oxidative stress in neuronal cells. Cell Death Discov.

[77] AhYoung AP, Jiang J, Zhang J, Khoi Dang X, Loo JA, Zhou ZH (2015). Conserved smp domains of the ermes complex bind phospholipids and mediate tether assembly. Proc Natl Acad Sci U S A.

[78] Wu W, Li W, Chen H, Jiang L, Zhu R, Feng D (2016). Fundc1 is a novel mitochondrial-associated-membrane (mam) protein required for hypoxia-induced mitochondrial fission and mitophagy. Autophagy.

[79] Gomez-Suaga P, Paillusson S, Stoica R, Noble W, Hanger DP, Miller CCJ (2017). The er-mitochondria tethering complex vapb-ptpip51 regulates autophagy. Curr Biol.

[80] Jeong H, Park J, Lee C (2016). Crystal structure of mdm12 reveals the architecture and dynamic organization of the ermes complex. EMBO Rep.

[81] Jeong H, Park J, Jun Y, Lee C (2017). Crystal structures of mmm1 and mdm12-mmm1 reveal mechanistic insight into phospholipid trafficking at er-mitochondria contact sites. Proc Natl Acad Sci U S A.

[82] Kornmann B, Osman C, Walter P (2011). The conserved gtpase gem1 regulates endoplasmic reticulum-mitochondria connections. Proc Natl Acad Sci U S A.

[83] Hirabayahi Y, Kwon SK, Paek H, Pernice WM, Paul MA, Lee J (2017). Er-mitochondria tethering by pdzd8 regulates ca2+ dynamics in mammalian neurons. Science.

[84] Kwak C, Shin S, Park JS, Jung M, Nhung TTM, Kang MG (2020). Contact-id, a tool for profiling organelle contact sites, reveals regulatory proteins of mitochondrial-associated membrane formation. Proc Natl Acad Sci U S A.

[85] Szabadkai G, Bianchi K, Várnai P, De Stefani D, Wieckowski MR, Cavagna D (2006). Chaperone-mediated coupling of endoplasmic reticulum and mitochondrial ca2+channels. J Cell Biol.

[86] Hayashi T, Su T-P (2007). Sigma-1 receptor chaperones at the er- mitochondrion interface regulate ca2+ signaling and cell survival. Cell.

[87] Watanabe S, Ilieva H, Tamada H, Nomura H, Komine O, Endo F (2016). Mitochondria-associated membrane collapse is a common pathomechanism in sigmar1- and sod1-linked als. EMBO Mol Med.

[88] Lepelletier FX, Vandesquille M, Asselin MC, Prenant C, Robinson AC, Mann DMA (2020). Evaluation of f-18-iam6067 as a sigma-1 receptor pet tracer for neurodegeneration in vivo in rodents and in human tissue. Theranostics.

[89] Park SJ, Lee SB, Suh Y, Kim SJ, Lee N, Hong JH (2017). Disc1 modulates neuronal stress responses by gate-keeping er-mitochondria ca(2+) transfer through the mam. Cell Rep.

[90] Bonneau B, Ando H, Kawaai K, Hirose M, Takahashi-Iwanaga H, Mikoshiba K (2016). Irbit controls apoptosis by interacting with the bcl-2 homolog, bcl2l10, and by promoting er-mitochondria contact. Elife.

[91] Thoudam T, Ha CM, Leem J, Chanda D, Park JS, Kim HJ (2019). Pdk4 augments er-mitochondria contact to dampen skeletal muscle insulin signaling during obesity. Diabetes.

[92] D'Eletto M, Rossin F, Occhigrossi L, Farrace MG, Faccenda D, Desai R (2018). Transglutaminase type 2 regulates er-mitochondria contact sites by interacting with grp75. Cell Rep.

[93] Matsuzaki H, Fujimoto T, Tanaka M, Shirasawa S (2013). Tespa1 is a novel component of mitochondria-associated endoplasmic reticulum membranes and affects mitochondrial calcium flux. Biochem Biophys Res Commun.

[94] Bonifati V, Rizzu P, van Baren MJ, Schaap O, Breedveld GJ, Krieger E (2003). Mutations in the dj-1 gene associated with autosomal recessive early-onset parkinsonism. Science.

[95] Zhou J, Zhang L, Wang M, Zhou L, Feng XP, Yu LL (2019). Cpx targeting dj-1 triggers ros-induced cell death and protective autophagy in colorectal cancer. Theranostics.

[96] Ottolini D, Cali T, Negro A, Brini M (2013). The parkinson disease-related protein dj-1 counteracts mitochondrial impairment induced by the tumour suppressor protein p53 by enhancing endoplasmic reticulum-mitochondria tethering. Hum Mol Genet.

[97] Naon D, Zaninello M, Giacomello M, Varanita T, Grespi F, Lakshminaranayan S (2016). Critical reappraisal confirms that mitofusin 2 is an endoplasmic reticulum-mitochondria tether. Proc Natl Acad Sci U S A.

[98] de Brito OM, Scorrano L (2008). Mitofusin 2 tethers endoplasmic reticulum to mitochondria. Nature.

[99] Basso V, Marchesan E, Peggion C, Chakraborty J, von Stockum S, Giacomello M (2018). Regulation of er-mitochondria contacts by parkin via mfn2. Pharmacol Res.

[100] Sugiura A, Nagashima S, Tokuyama T, Amo T, Matsuki Y, Ishido S (2013). Mitol regulates endoplasmic reticulum-mitochondria contacts via mitofusin2. Mol Cell.

[101] Filadi R, Greotti E, Turacchio G, Luini A, Pozzan T, Pizzo P (2015). Mitofusin 2 ablation increases endoplasmic reticulum-mitochondria coupling. Proc Natl Acad Sci U S A.

[102] Leal NS, Schreiner B, Pinho CM, Filadi R, Wiehager B, Karlstrom H (2016). Mitofusin-2 knockdown increases er-mitochondria contact and decreases amyloid beta-peptide production. J Cell Mol Med.

[103] He C, Klionsky DJ (2009). Regulation mechanisms and signaling pathways of autophagy. Annu Rev Genet.

[104] Wang N, Tan HY, Li S, Feng YB (2017). Atg9b deficiency suppresses autophagy and potentiates endoplasmic reticulum stress-associated hepatocyte apoptosis in hepatocarcinogenesis. Theranostics.

[105] Li H, Liu J, Cao WJ, Xiao XJ, Liang L, Liu-Smith F (2019). C-myc/mir-150/epg5 axis mediated dysfunction of autophagy promotes development of non-small cell lung cancer. Theranostics.

[106] Liu L, Feng D, Chen G, Chen M, Zheng Q, Song P (2012). Mitochondrial outer-membrane protein fundc1 mediates hypoxia-induced mitophagy in mammalian cells. Nat Cell Biol.

[107] Wu S, Lu Q, Wang Q, Ding Y, Ma Z, Mao X (2017). Binding of fun14 domain containing 1 with inositol 1,4,5-trisphosphate receptor in mitochondria-associated endoplasmic reticulum membranes maintains mitochondrial dynamics and function in hearts in vivo. Circulation.

[108] Wu W, Lin C, Wu K, Jiang L, Wang X, Li W (2016). Fundc1 regulates mitochondrial dynamics at the er-mitochondrial contact site under hypoxic conditions. EMBO J.

[109] Lv BF, Yu CF, Chen YY, Lu Y, Guo JH, Song QS (2006). Protein tyrosine phosphatase interacting protein 51 (ptpip51) is a novel mitochondria protein with an n-terminal mitochondrial targeting sequence and induces apoptosis. Apoptosis.

[110] De Vos KJ, Morotz GM, Stoica R, Tudor EL, Lau KF, Ackerley S (2012). Vapb interacts with the mitochondrial protein ptpip51 to regulate calcium homeostasis. Hum Mol Genet.

[111] Stoica R, De Vos KJ, Paillusson S, Mueller S, Sancho RM, Lau KF (2014). Er-mitochondria associations are regulated by the vapb-ptpip51 interaction and are disrupted by als/ftd-associated tdp-43. Nat Commun.

[112] Stoica R, Paillusson S, Gomez-Suaga P, Mitchell JC, Lau DH, Gray EH (2016). Als/ftd-associated fus activates gsk-3beta to disrupt the vapb-ptpip51 interaction and er-mitochondria associations. EMBO Rep.

[113] Le Vasseur M, Chen VC, Huang K, Vogl WA, Naus CC (2019). Pannexin 2 localizes at er-mitochondria contact sites. Cancers (Basel).

[114] Gilady SY, Bui M, Lynes EM, Benson MD, Watts R, Vance JE (2010). Ero1alpha requires oxidizing and normoxic conditions to localize to the mitochondria-associated membrane (mam). Cell Stress Chaperones.

[115] Cho IT, Adelmant G, Lim Y, Marto JA, Cho G, Golden JA (2017). Ascorbate peroxidase proximity labeling coupled with biochemical fractionation identifies promoters of endoplasmic reticulum-mitochondrial contacts. J Biol Chem.

[116] Verfaillie T, Rubio N, Garg AD, Bultynck G, Rizzuto R, Decuypere JP (2012). Perk is required at the er-mitochondrial contact sites to convey apoptosis after ros-based er stress. Cell Death Differ.

[117] Raturi A, Gutierrez T, Ortiz-Sandoval C, Ruangkittisakul A, Herrera-Cruz MS, Rockley JP (2016). Tmx1 determines cancer cell metabolism as a thiol-based modulator of er-mitochondria ca2+ flux. J Cell Biol.

[118] Lynes EM, Bui M, Yap MC, Benson MD, Schneider B, Ellgaard L (2012). Palmitoylated tmx and calnexin target to the mitochondria-associated membrane. EMBO J.

[119] Demetriadou A, Morales-Sanfrutos J, Nearchou M, Baba O, Kyriacou K, Tate EW (2017). Mouse stbd1 is n-myristoylated and affects er-mitochondria association and mitochondrial morphology. J Cell Sci.

[120] Filadi R, Leal NS, Schreiner B, Rossi A, Dentoni G, Pinho CM (2018). Tom70 sustains cell bioenergetics by promoting ip3r3-mediated er to mitochondria ca(2+) transfer. Curr Biol.

[121] Elbaz-Alon Y, Eisenberg-Bord M, Shinder V, Stiller SB, Shimoni E, Wiedemann N (2015). Lam6 regulates the extent of contacts between organelles. Cell Rep.

[122] Murley A, Sarsam RD, Toulmay A, Yamada J, Prinz WA, Nunnari J (2015). Ltc1 is an er-localized sterol transporter and a component of er-mitochondria and er-vacuole contacts. J Cell Biol.

[123] Tabara LC, Escalante R (2016). Vmp1 establishes er-microdomains that regulate membrane contact sites and autophagy. PLoS One.

[124] Bravo-Sagua R, Parra V, Ortiz-Sandoval C, Navarro-Marquez M, Rodriguez AE, Diaz-Valdivia N (2019). Caveolin-1 impairs pka-drp1-mediated remodelling of er-mitochondria communication during the early phase of er stress. Cell Death Differ.

[125] Missiroli S, Bonora M, Patergnani S, Giorgi C (2017). Novel function of the tumor suppressor pml at er-mitochondria sites in the control of autophagy. Oncotarget.

[126] Gelmetti V, De Rosa P, Torosantucci L, Marini ES, Romagnoli A, Di Rienzo M (2017). Pink1 and becn1 relocalize at mitochondria-associated membranes during mitophagy and promote er-mitochondria tethering and autophagosome formation. Autophagy.

[127] Cho KF, Branon TC, Rajeev S, Svinkina T, Udeshi ND, Thoudam T (2020). Split-turboid enables contact-dependent proximity labeling in cells. Proc Natl Acad Sci U S A.

[128] Rossi A, Pizzo P, Filadi R (2019). Calcium, mitochondria and cell metabolism: A functional triangle in bioenergetics. Biochim Biophys Acta Mol Cell Res.

[129] Cali T, Ottolini D, Negro A, Brini M (2013). Enhanced parkin levels favor er-mitochondria crosstalk and guarantee ca2+ transfer to sustain cell bioenergetics. Biochim Biophys Acta Mol Basis Dis.

[130] Guardia-Laguarta C, Area-Gomez E, Rub C, Liu YH, Magrane J, Becker D (2014). Alpha-synuclein is localized to mitochondria-associated er membranes. J Neurosci.

[131] Hedskog L, Pinho CM, Filadi R, Ronnback A, Hertwig L, Wiehager B (2013). Modulation of the endoplasmic reticulum-mitochondria interface in alzheimer's disease and related models. Proc Natl Acad Sci U S A.

[132] Cheung KH, Shineman D, Müller M, Cárdenas C, Mei L, Yang J (2008). Mechanism of ca2+ disruption in alzheimer's disease by presenilin regulation of insp3 receptor channel gating. Neuron.

[133] Lim Y, Cho IT, Schoel LJ, Cho G, Golden JA (2015). Hereditary spastic paraplegia-linked reep1 modulates endoplasmic reticulum/mitochondria contacts. Ann Neurol.

[134] Rouzier C, Moore D, Delorme C, Lacas-Gervais S, Ait-El-Mkadem S, Fragaki K (2017). A novel cisd2 mutation associated with a classical wolfram syndrome phenotype alters ca2+ homeostasis and er-mitochondria interactions. Hum Mol Genet.

[135] Sutendra G, Dromparis P, Wright P, Bonnet S, Haromy A, Hao ZR (2011). The role of nogo and the mitochondria-endoplasmic reticulum unit in pulmonary hypertension. Sci Transl Med.

[136] Tubbs E, Chanon S, Robert M, Bendridi N, Bidaux G, Chauvin MA (2018). Disruption of mitochondria-associated endoplasmic reticulum membrane (mam) integrity contributes to muscle insulin resistance in mice and humans. Diabetes.

[137] Mellman I, Warren G (2000). The road taken: Past and future foundations of membrane traffic. Cell.

[138] Boncompain G, Weigel AV (2018). Transport and sorting in the golgi complex: Multiple mechanisms sort diverse cargo. Curr Opin Cell Biol.

[139] Wilson C, Venditti R, Rega LR, Colanzi A, D'Angelo G, De Matteis MA (2011). The golgi apparatus: An organelle with multiple complex functions. Biochem J.

[140] Nakamura N (2010). Emerging new roles of gm130, a cis-golgi matrix protein, in higher order cell functions. J Pharmacol Sci.

[141] Geng J, Klionsky DJ (2010). The golgi as a potential membrane source for autophagy. Autophagy.

[142] Brandizzi F, Barlowe C (2013). Organization of the er-golgi interface for membrane traffic control. Nat Rev Mol Cell Biol.

[143] Ben-Tekaya H, Miura K, Pepperkok R, Hauri HP (2005). Live imaging of bidirectional traffic from the ergic. J Cell Sci.

[144] Novikoff PM, Novikoff AB, Quintana N, Hauw JJ (1971). Golgi apparatus, gerl, and lysosomes of neurons in rat dorsal root ganglia, studied by thick section and thin section cytochemistry. J Cell Biol.

[145] Rambourg A, Clermont Y, Hermo L (1979). Three-dimensional architecture of the golgi apparatus in sertoli cells of the rat. Am J Anat.

[146] Venditti R, Rega LR, Masone MC, Santoro M, Polishchuk E, Sarnataro D (2019). Molecular determinants of er-golgi contacts identified through a new fret-flim system. J Cell Biol.

[147] Peretti D, Dahan N, Shimoni E, Hirschberg K, Lev S (2008). Coordinated lipid transfer between the endoplasmic reticulum and the golgi complex requires the vap proteins and is essential for golgi-mediated transport. Mol Biol Cell.

[148] Pietrangelo A, Ridgway ND (2018). Golgi localization of oxysterol binding protein-related protein 4l (orp4l) is regulated by ligand binding. J Cell Sci.

[149] Mesmin B, Bigay J, Moser von Filseck J, Lacas-Gervais S, Drin G, Antonny B (2013). A four-step cycle driven by pi(4)p hydrolysis directs sterol/pi(4)p exchange by the er-golgi tether osbp. Cell.

[150] Jamecna D, Polidori J, Mesmin B, Dezi M, Levy D, Bigay J (2019). An intrinsically disordered region in osbp acts as an entropic barrier to control protein dynamics and orientation at membrane contact sites. Dev Cell.

[151] Liu LK, Choudhary V, Toulmay A, Prinz WA (2017). An inducible er-golgi tether facilitates ceramide transport to alleviate lipotoxicity. J Cell Biol.

[152] Mullen TD, Obeid LM (2012). Ceramide and apoptosis: Exploring the enigmatic connections between sphingolipid metabolism and programmed cell death. Anticancer Agents Med Chem.

[153] Huitema K, van den Dikkenberg J, Brouwers JFHM, Holthuis JCM (2004). Identification of a family of animal sphingomyelin synthases. EMBO J.

[154] Hussain MM, Jin WJ, Jiang XC (2012). Mechanisms involved in cellular ceramide homeostasis. Nutr Metab.

[155] Kawano M, Kumagai K, Nishijima M, Hanada K (2006). Efficient trafficking of ceramide from the endoplasmic reticulum to the golgi apparatus requires a vamp-associated protein-interacting ffat motif of cert. J Biol Chem.

[156] Deng YQ, Pakdel M, Blank B, Sundberg EL, Burd CG, von Blume J (2018). Activity of the spca1 calcium pump couples sphingomyelin synthesis to sorting of secretory proteins in the trans-golgi network. Dev Cell.

[157] Mu FT, Callaghan JM, Steele-Mortimer O, Stenmark H, Parton RG, Campbell PL (1995). Eea1, an early endosome-associated protein. Eea1 is a conserved alpha-helical peripheral membrane protein flanked by cysteine "fingers" and contains a calmodulin-binding iq motif. J Biol Chem.

[158] Chavrier P, Parton RG, Hauri HP, Simons K, Zerial M (1990). Localization of low molecular weight gtp binding proteins to exocytic and endocytic compartments. Cell.

[159] Zhang JX, Zhang XD, Liu G, Chang DF, Liang X, Zhu XB (2016). Intracellular trafficking network of protein nanocapsules: Endocytosis, exocytosis and autophagy. Theranostics.

[160] Feng Y, Press B, Wandinger-Ness A (1995). Rab 7: An important regulator of late endocytic membrane traffic. J Cell Biol.

[161] Fader CM, Colombo MI (2009). Autophagy and multivesicular bodies: Two closely related partners. Cell Death Differ.

[162] Li Y, Zhao Y, Hu J, Xiao J, Qu L, Wang Z (2014). A novel er-localized transmembrane protein, emc6, interacts with rab5a and regulates cell autophagy. Autophagy.

[163] Hoyer MJ, Chitwood PJ, Ebmeier CC, Striepen JF, Qi RZ, Old WM (2018). A novel class of er membrane proteins regulates er-associated endosome fission. Cell.

[164] Eden ER, White IJ, Tsapara A, Futter CE (2010). Membrane contacts between endosomes and er provide sites for ptp1b-epidermal growth factor receptor interaction. Nat Cell Biol.

[165] Friedman JR, DiBenedetto JR, West M, Rowland AA, Voeltz GK, Hegde RS (2013). Endoplasmic reticulum-endosome contact increases as endosomes traffic and mature. Mol Biol Cell.

[166] Rowland Ashley A, Chitwood Patrick J, Phillips Melissa J, Voeltz Gia K (2014). Er contact sites define the position and timing of endosome fission. Cell.

[167] Su WC, Chao TC, Huang YL, Weng SC, Jeng KS, Lai MMC (2011). Rab5 and class iii phosphoinositide 3-kinase vps34 are involved in hepatitis c virus ns4b-induced autophagy. J Virol.

[168] Wells A, Kassis J, Solava J, Turner T, Lauffenburger DA (2002). Growth factor-induced cell motility in tumor invasion. Acta Oncol.

[169] Löf-Öhlin ZM, Nyeng P, Bechard ME, Hess K, Bankaitis E, Greiner TU (2017). Egfr signalling controls cellular fate and pancreatic organogenesis by regulating apicobasal polarity. Nat Cell Biol.

[170] Sordella R, Bell DW, Haber DA, Settleman J (2004). Gefitinib-sensitizing egfr mutations in lung cancer activate anti-apoptotic pathways. Science.

[171] Fu WY, Sun HF, Zhao Y, Chen MT, Yang XL, Liu Y (2019). Bcap31 drives tnbc development by modulating ligand-independent egfr trafficking and spontaneous egfr phosphorylation. Theranostics.

[172] Eden Emily R, Burgoyne T, Edgar James R, Sorkin A, Futter Clare E (2012). The relationship between er-multivesicular body membrane contacts and the escrt machinery. Biochem Soc Trans.

[173] Haj FG, Verveer PJ, Squire A, Neel BG, Bastiaens PI (2002). Imaging sites of receptor dephosphorylation by ptp1b on the surface of the endoplasmic reticulum. Science.

[174] Rety S, Osterloh D, Arie JP, Tabaries S, Seeman J, Russo-Marie F (2000). Structural basis of the ca(2+)-dependent association between s100c (s100a11) and its target, the n-terminal part of annexin i. Structure.

[175] Eden Emily R, Sanchez-Heras E, Tsapara A, Sobota A, Levine Tim P, Futter Clare E (2016). Annexin a1 tethers membrane contact sites that mediate er to endosome cholesterol transport. Dev Cell.

[176] Dong J, Du X, Wang H, Wang J, Lu C, Chen X (2019). Allosteric enhancement of orp1-mediated cholesterol transport by pi(4,5)p2/pi(3,4)p2. Nat Commun.

[177] Waterman-Storer CM, Karki S, Holzbaur EL (1995). The p150glued component of the dynactin complex binds to both microtubules and the actin-related protein centractin (arp-1). Proc Natl Acad Sci U S A.

[178] Johansson M, Rocha N, Zwart W, Jordens I, Janssen L, Kuijl C (2007). Activation of endosomal dynein motors by stepwise assembly of rab7-rilp-p150glued, orp1l, and the receptor betalll spectrin. J Cell Biol.

[179] Rocha N, Kuijl C, van der Kant R, Janssen L, Houben D, Janssen H (2009). Cholesterol sensor orp1l contacts the er protein vap to control rab7-rilp-p150gluedand late endosome positioning. J Cell Biol.

[180] van der Kant R, Fish A, Janssen L, Janssen H, Krom S, Ho N (2013). Late endosomal transport and tethering are coupled processes controlled by rilp and the cholesterol sensor orp1l. J Cell Sci.

[181] Shirane M, Nakayama KI (2006). Protrudin induces neurite formation by directional membrane trafficking. Science.

[182] Raiborg C, Wenzel EM, Pedersen NM, Olsvik H, Schink KO, Schultz SW (2015). Repeated er-endosome contacts promote endosome translocation and neurite outgrowth. Nature.

[183] Matsuzaki F, Shirane M, Matsumoto M, Nakayama KI (2011). Protrudin serves as an adaptor molecule that connects kif5 and its cargoes in vesicular transport during process formation. Mol Biol Cell.

[184] Krauss M, Haucke V (2015). A grab to move on: Er-endosome contacts in membrane protrusion formation and neurite outgrowth. EMBO J.

[185] Alpy F, Tomasetto C (2014). Start ships lipids across interorganelle space. Biochimie.

[186] Wilhelm LP, Wendling C, Vedie B, Kobayashi T, Chenard MP, Tomasetto C (2017). Stard3 mediates endoplasmic reticulum-to-endosome cholesterol transport at membrane contact sites. EMBO J.

[187] Wijdeven RH, Jongsma ML, Neefjes J, Berlin I (2015). Er contact sites direct late endosome transport. Bioessays.

[188] Thiam AR, Beller M (2017). The why, when and how of lipid droplet diversity. J Cell Sci.

[189] Hariri H, Rogers S, Ugrankar R, Liu YL, Feathers JR, Henne WM (2017). Lipid droplet biogenesis is spatially coordinated at er-vacuole contacts under nutritional stress. EMBO Rep.

[190] Nguyen TB, Olzmann JA (2019). Getting a handle on lipid droplets: Insights into er-lipid droplet tethering. J Cell Biol.

[191] Hariri H, Speer N, Bowerman J, Rogers S, Fu G, Reetz E (2019). Mdm1 maintains endoplasmic reticulum homeostasis by spatially regulating lipid droplet biogenesis. J Cell Biol.

[192] Szymanski KM, Binns D, Bartz R, Grishin NV, Li WP, Agarwal AK (2007). The lipodystrophy protein seipin is found at endoplasmic reticulum lipid droplet junctions and is important for droplet morphology. Proc Natl Acad Sci U S A.

[193] Fei W, Shui G, Gaeta B, Du X, Kuerschner L, Li P (2008). Fld1p, a functional homologue of human seipin, regulates the size of lipid droplets in yeast. J Cell Biol.

[194] Wolinski H, Hofbauer HF, Hellauer K, Cristobal-Sarramian A, Kolb D, Radulovic M (2015). Seipin is involved in the regulation of phosphatidic acid metabolism at a subdomain of the nuclear envelope in yeast. Biochim Biophys Acta.

[195] Salo VT, Belevich I, Li S, Karhinen L, Vihinen H, Vigouroux C (2016). Seipin regulates er-lipid droplet contacts and cargo delivery. EMBO J.

[196] Salo VT, Li S, Vihinen H, Holtta-Vuori M, Szkalisity A, Horvath P (2019). Seipin facilitates triglyceride flow to lipid droplet and counteracts droplet ripening via endoplasmic reticulum contact. Dev Cell.

[197] Kassan A, Herms A, Fernandez-Vidal A, Bosch M, Schieber NL, Reddy BJN (2013). Acyl-coa synthetase 3 promotes lipid droplet biogenesis in er microdomains. J Cell Biol.

[198] Magre J, Delepine M, Khallouf E, Gedde-Dahl T, Van Maldergem L, Sobel E (2001). Identification of the gene altered in berardinelli-seip congenital lipodystrophy on chromosome 11q13. Nat Genet.

[199] Ozeki S, Cheng J, Tauchi-Sato K, Hatano N, Taniguchi H, Fujimoto T (2005). Rab18 localizes to lipid droplets and induces their close apposition to the endoplasmic reticulum-derived membrane. J Cell Sci.

[200] Xu D, Li Y, Wu L, Li Y, Zhao D, Yu J (2018). Rab18 promotes lipid droplet (ld) growth by tethering the er to lds through snare and nrz interactions. J Cell Biol.

[201] Hirose H, Arasaki K, Dohmae N, Takio K, Hatsuzawa K, Nagahama M (2004). Implication of zw10 in membrane trafficking between the endoplasmic reticulum and golgi. EMBO J.

[202] Itakura E, Mizushima N (2010). Characterization of autophagosome formation site by a hierarchical analysis of mammalian atg proteins. Autophagy.

[203] Gao G, Sheng Y, Yang H, Chua BT, Xu L (2019). Dfcp1 associates with lipid droplets. Cell Biol Int.

[204] Li D, Zhao YG, Li D, Zhao H, Huang J, Miao G (2019). The er-localized protein dfcp1 modulates er-lipid droplet contact formation. Cell Rep.

[205] Xu N, Zhang SO, Cole RA, McKinney SA, Guo F, Haas JT (2012). The fatp1-dgat2 complex facilitates lipid droplet expansion at the er-lipid droplet interface. J Cell Biol.

[206] Weber-Boyvat M, Kentala H, Peranen J, Olkkonen VM (2015). Ligand-dependent localization and function of orp-vap complexes at membrane contact sites. Cell Mol Life Sci.

[207] Zhao YG, Chen Y, Miao GY, Zhao HY, Qu WY, Li DF (2017). The er-localized transmembrane protein epg-3/vmp1 regulates serca activity to control er-isolation membrane contacts for autophagosome formation. Mol Cell.

[208] Shibata Y, Voeltz GK, Rapoport TA (2006). Rough sheets and smooth tubules. Cell.

[209] Hoffman AM, Chen Q, Zheng TL, Nicchitta CV (2019). Heterogeneous translational landscape of the endoplasmic reticulum revealed by ribosome proximity labeling and transcriptome analysis. J Biol Chem.

[210] Connolly T, Gilmore R (1989). The signal recognition particle receptor mediates the gtp-dependent displacement of srp from the signal sequence of the nascent polypeptide. Cell.

[211] Wu WC, Liu HW, Lin A (2007). Human ribosomal protein l7 displays an er binding property and is involved in ribosome-er association. FEBS Lett.

[212] Grossmann D, Berenguer-Escuder C, Bellet ME, Scheibner D, Bohler J, Massart F (2019). Mutations in rhot1 disrupt endoplasmic reticulum-mitochondria contact sites interfering with calcium homeostasis and mitochondrial dynamics in parkinson's disease. Antioxid Redox Signal.

[213] Zampese E, Fasolato C, Kipanyula MJ, Bortolozzi M, Pozzan T, Pizzo P (2011). Presenilin 2 modulates endoplasmic reticulum (er)-mitochondria interactions and ca2+ cross-talk. Proc Natl Acad Sci U S A.

[214] Kumar N, Leonzino M, Hancock-Cerutti W, Horenkamp FA, Li P, Lees JA (2018). Vps13a and vps13c are lipid transport proteins differentially localized at er contact sites. J Cell Biol.

